# Multiple Regulatory Systems Coordinate DNA Replication with Cell Growth in *Bacillus subtilis*

**DOI:** 10.1371/journal.pgen.1004731

**Published:** 2014-10-23

**Authors:** Heath Murray, Alan Koh

**Affiliations:** Centre for Bacterial Cell Biology, Institute for Cell and Molecular Biosciences, Newcastle University, Newcastle Upon Tyne, United Kingdom; A*STAR, Singapore

## Abstract

In many bacteria the rate of DNA replication is linked with cellular physiology to ensure that genome duplication is coordinated with growth. Nutrient-mediated growth rate control of DNA replication initiation has been appreciated for decades, however the mechanism(s) that connects these cell cycle activities has eluded understanding. In order to help address this fundamental question we have investigated regulation of DNA replication in the model organism *Bacillus subtilis*. Contrary to the prevailing view we find that changes in DnaA protein level are not sufficient to account for nutrient-mediated growth rate control of DNA replication initiation, although this regulation does require both DnaA and the endogenous replication origin. We go on to report connections between DNA replication and several essential cellular activities required for rapid bacterial growth, including respiration, central carbon metabolism, fatty acid synthesis, phospholipid synthesis, and protein synthesis. Unexpectedly, the results indicate that multiple regulatory systems are involved in coordinating DNA replication with cell physiology, with some of the regulatory systems targeting *oriC* while others act in a *oriC*-independent manner. We propose that distinct regulatory systems are utilized to control DNA replication in response to diverse physiological and chemical changes.

## Author Summary

DNA replication must be coordinated with cellular physiology to ensure proper genome inheritance. Model bacteria such as the soil-dwelling *Bacillus subtilis* can achieve a wide range of growth rates in response to nutritional and chemical signals. In order to match the rate of DNA synthesis to the rate of nutrient-mediated cell growth, bacteria regulate the initiation frequency of DNA replication. This control of bacterial DNA replication initiation was first observed over forty years ago, however the molecular basis for this regulation has remained hotly debated. In this paper we test one of the leading models for nutrient-mediated growth rate regulation in bacteria, namely that the abundance of the master DNA replication initiation protein DnaA dictates the frequency of DNA replication events. Critically, our results show that changes in DnaA protein level are not sufficient to account for nutrient-mediated growth rate regulation of DNA replication initiation in *B. subtilis*. We then go on to show that there are strong connections between DNA replication and several essential cellular activities, which unexpectedly indicates that there is likely more than one single regulatory pathway involved in coordinating DNA replication with cell physiology. We believe that our work changes thinking regarding this long-standing biological question and reinvigorates the search for the molecular basis of these critical regulatory systems.

## Introduction

DNA replication must be coordinated with the cell cycle to ensure proper genome inheritance. For many bacteria cellular physiology dictates the rate of growth and division. In nutrient-rich media that support rapid growth rates, bacteria synthesize DNA more rapidly by increasing the frequency of DNA replication initiation [1]–[3]. This control system is termed nutrient-mediated growth rate regulation and although it has been appreciated for decades, the molecular mechanisms that connect cell physiology with DNA replication initiation remain debatable.

Historically it has been thought that there is a constant cell mass or cell size at the time of bacterial DNA replication initiation and it has been proposed that a positive regulator would accumulate in a growth-dependent manner to trigger DNA replication initiation when cells attained a critical size [4]. However, modern quantitative analysis of single bacterial cells within steady-state populations has shown that the relationship between DNA replication initiation and cell mass is variable, indicating that the control for timing of DNA replication initiation is not governed by a direct connection with mass accumulation [5].

DnaA is the master bacterial DNA replication initiator protein and is a candidate factor to connect cell physiology with DNA synthesis. DnaA is a member of the AAA+ family of ATPases and shares homology with archaeal and eukaryotic initiator proteins. DnaA directly stimulates DNA replication initiation from a single defined origin of replication (*oriC*) once per cell cycle. Multiple ATP-bound DnaA molecules bind to an array of recognition sequences (DnaA-box 5′-TTATCCACA-3′) within *oriC* where they assemble into a helical filament that promotes duplex DNA unwinding [6], [7].

Studies in *Escherichia coli* have suggested that the amount of ATP-bound DnaA dictates the rate of DNA replication initiation. Artificial overexpression of DnaA increases the frequency of DNA replication initiation [8], [9]. Conversely, decreasing the amount of DnaA per cell by synthetically promoting early cell division delays DNA replication initiation and modest increases in DnaA levels alleviate this delay, supporting the view that growth-dependent accumulation of DnaA is the trigger for replication initiation in *E. coli* [10]. However, it remains uncertain whether the amount of ATP-bound DnaA is the primary regulator that coordinates DNA replication initiation with cell growth in wild-type *E. coli* cells [11].

In contrast to *E. coli*, studies in *Bacillus subtilis* have suggested that the amount of DnaA may not dictate the rate of DNA replication initiation. Artificially decreasing cell size by stimulating cell division (thereby lowering the amount DnaA per cell to ∼70% of wild-type) did not affect DNA replication initiation [10]. Moreover, results from overexpression of DnaA in *B. subtilis* are not clear. Increased expression of DnaA alone causes cell elongation, cell growth inhibition, and induction of the SOS DNA damage response due to depletion of DnaN because of autoregulation of the *dnaA-dnaN* operon by DnaA [12]. To circumvent this problem DnaA was co-overexpressed with DnaN, and under this condition DNA replication initiation does increase [12]. However, subsequent experiments demonstrated that overexpression of DnaN alone increases DNA replication initiation by repressing the activity of the regulatory protein YabA (an inhibitor of DnaA)[13], suggesting that this could account for the effect on DNA replication initiation when DnaA and DnaN were co-overexpressed.

In this study we have investigated nutrient-mediated growth rate control of DNA replication initiation in *B. subtilis*. We find that changes in DnaA protein level are not sufficient to account for nutrient-mediated growth rate regulation of DNA replication initiation, although this regulation does require both DnaA and *oriC*. We then present evidence suggesting that multiple regulatory systems are involved in coordinating DNA synthesis with cell physiology, and that depending on the nature of the growth limitation, control of DNA replication acts through either *oriC*-dependent or *oriC*-independent mechanisms.

## Results

### Changes in DnaA levels cannot account for nutrient-mediated growth rate regulation of DNA replication initiation in *B. subtilis*

**Figure 1:**
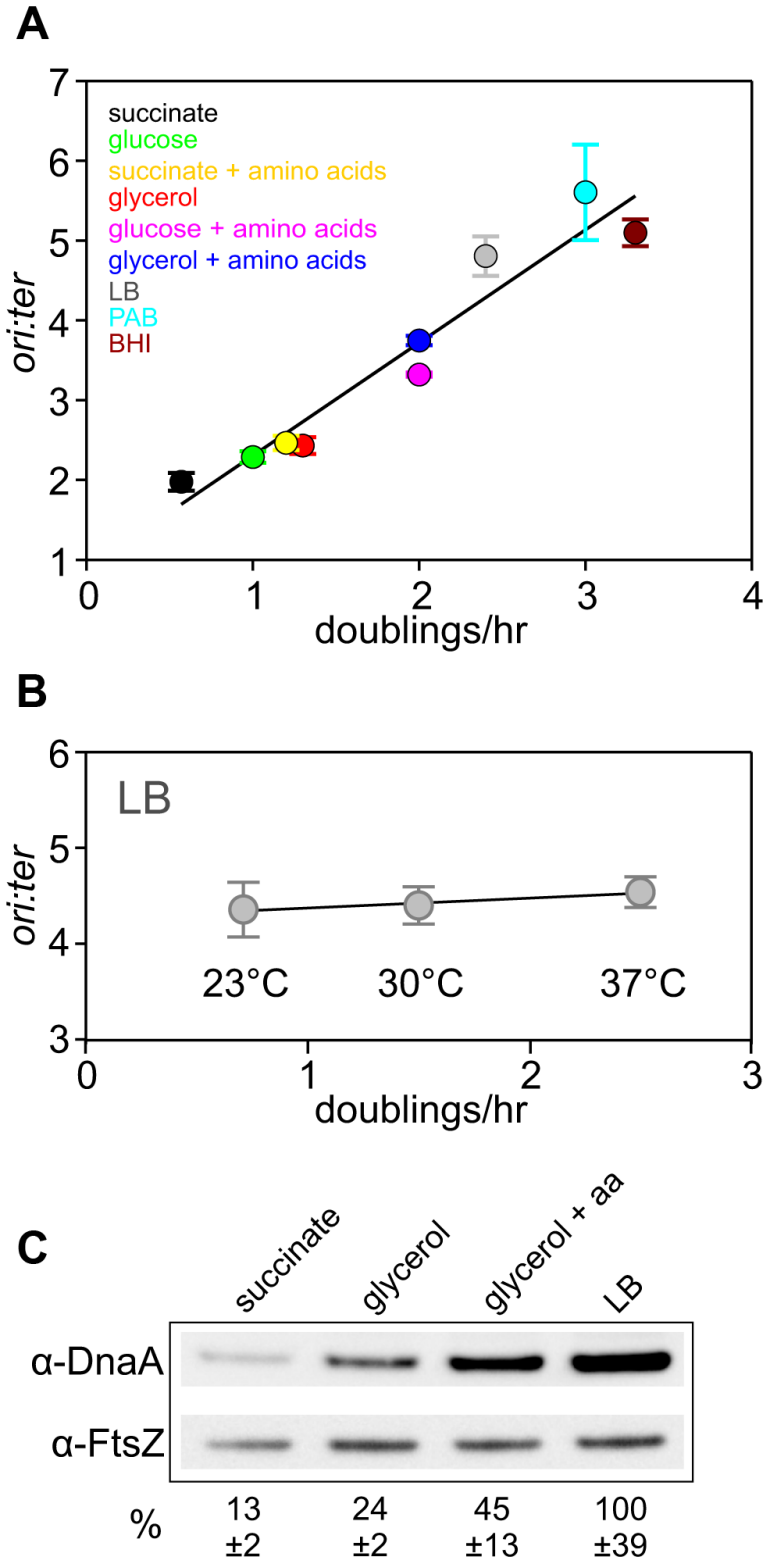
Nutrient-mediated growth rate regulation of DNA replication initiation in *B. subtilis*. (**A**) Culturing *B. subtilis* in a different media generates a range of steady-state growth rates and affects the frequency of DNA replication initiation. A wild-type strain (HM222) was grown overnight at 37°C in minimal media supplemented with succinate and amino acids (20 µg/ml). The culture was diluted 1∶100 into various media to generate a range of steady-state growth rates and grown at 37°C until an A_600_ of 0.3–0.4. Genomic DNA was harvested from cells and marker frequency analysis was determined using qPCR. The *ori:ter* ratios are plotted versus growth rate (error bars indicate the standard deviation of three technical replicates). Representative data are shown from a single experiment; independently performed experiments are shown in Figures 4 and S5. (**B**) Culturing *B. subtilis* at different temperatures generates a range of steady-state growth rates but does not affect the frequency of DNA replication initiation. A wild-type strain (HM715) was grown overnight at 23°C in LB. The culture was diluted 1∶100 into LB and incubated at different temperatures to generate a range of steady-state growth rates until an A_600_ of 0.2–0.3. Genomic DNA was harvested from cells and marker frequency analysis was determined using qPCR. The *ori:ter* ratios are plotted versus growth rate (error bars indicate the standard deviation of three technical replicates). Representative data are shown from a single experiment; an independently performed replicate of the experiment is shown in Figure S1. (**C**) Measurement of DnaA protein levels at various growth rates in wild-type *B. subtilis* (HM715). Cultures were grown at 37°C overnight as in (A) and diluted 1∶100 into various media (succinate, glycerol, glycerol + amino acids, LB). to generate a range of steady-state growth rates until an A_600_ of 0.6–0.8. Cells were lysed and DnaA protein was detected using Western blot analysis (FtsZ protein was likewise detected and used as a loading control). For each culture media the average amount of DnaA (+/− standard deviation) from at least three biological replicates was determined using densitometry; values were normalized to LB.

Steady-state bacterial growth rates can be manipulated by culturing cells in media that contain differing amounts of nutrients, with rich media supporting faster growth because resources are not required to synthesize cellular building blocks *de novo*. In response to different nutrient-mediated steady-state growth rates, bacteria control DNA synthesis by varying the frequency of DNA replication initiation while maintaining a constant speed of elongation [1]–[3], [14]. The rate of DNA replication initiation can be determined by marker frequency analysis (i.e. - measuring the ratio of DNA at the replication origin (*ori*) versus the replication terminus (*ter*) using quantitative PCR), and Figure 1A shows the positive correlation between DNA replication initiation and nutrient-mediated growth rates (cell doublings per hour measured using spectrophotometry). It is important to state that experimental approaches which change bacterial growth rates without altering the chemical composition of the cell (e.g. – varying temperature) do not influence the rate of DNA replication initiation (Figures 1B, S1; [15], [16]). Thus, varying nutrient availability modulates bacterial physiology, in turn affecting cell growth and DNA replication initiation [3].

It has been reported that DnaA protein level determines the frequency of DNA replication initiation in *E. coli* [8], [9], therefore we wondered whether the amount of DnaA could account for nutrient-mediated growth rate regulation of DNA replication initiation in *B. subtilis* [14]. Western blot analysis shows that DnaA concentration increases with faster steady-state growth rates (Figure 1C; the tubulin homolog FtsZ was used as a loading control because its concentration is growth-rate independent [17],[18]). Since *B. subtilis* cell size increases as a function of growth rate, the number of DnaA molecules would also be greater in larger cells formed during fast growth (Figure S2)[14]. This conclusion is in agreement with absolute quantification of DnaA proteins per cell determined at different growth rates using mass spectrometry (163-337 molecules at 0.5 doublings/hr; 875-1791 molecules at 1.0 doublings/hr)[18]. These results indicate that the amount of DnaA protein could account for nutrient-mediated growth rate regulation of DNA replication initiation in *B. subtilis*.

**Figure 2:**
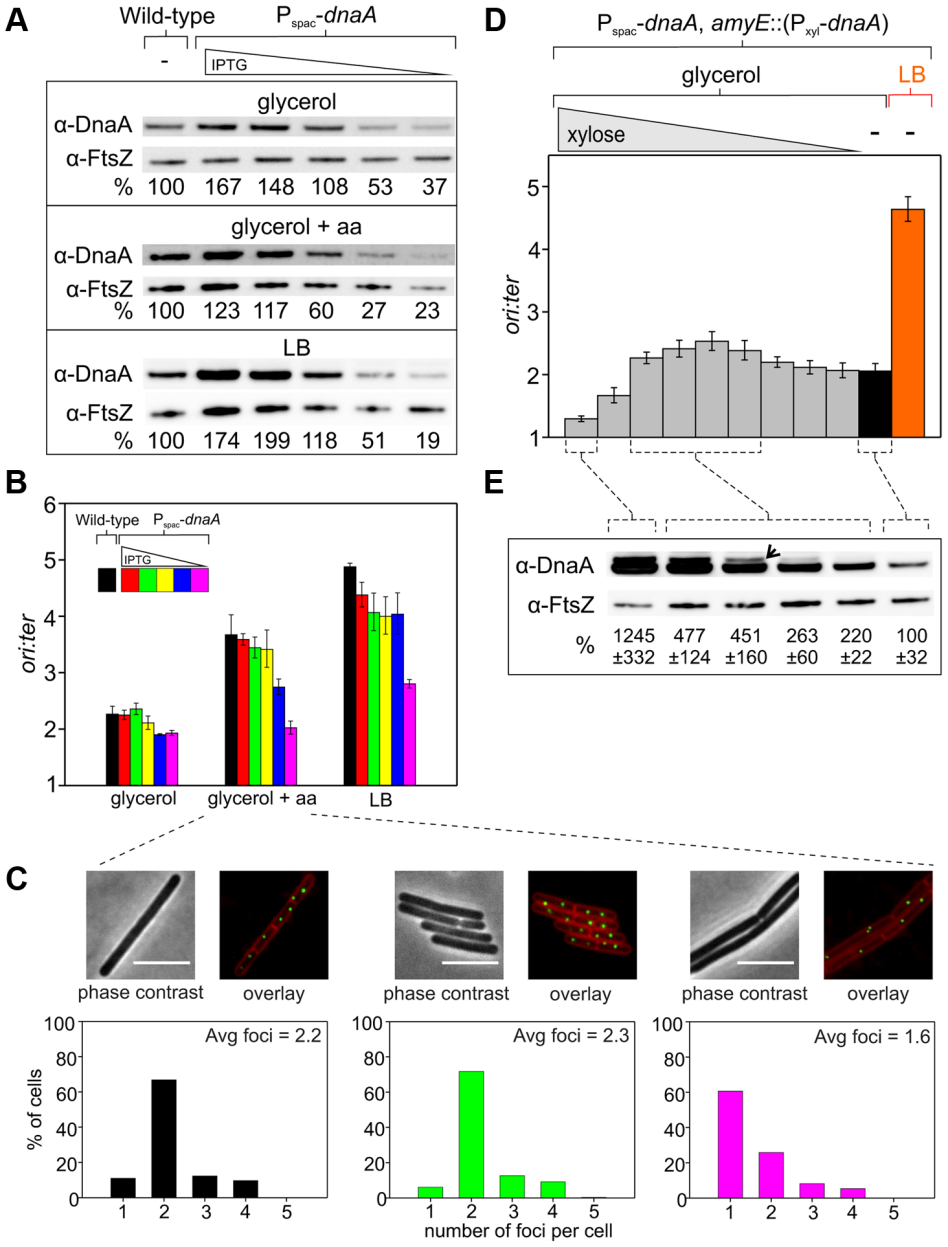
Changes in DnaA protein level are not sufficient to account for nutrient-mediated growth rate regulation of DNA replication initiation in *B. subtilis.* (**A**) The endogenous *dnaA* gene was placed under the control of the IPTG-inducible promoter P_spac_ to generate a range of DnaA protein levels. Strains were grown overnight at 37°C in minimal media supplemented with succinate and amino acids (20 µg/ml); IPTG (400 µM) and erythromycin was added to HM742. The cultures were diluted 1∶100 into various media (glycerol, glycerol + amino acids, LB) to generate a range of steady-state growth rates and grown at 37°C until an A_600_ of 0.5–0.6; in each medium HM742 was supplemented with erythromycin and a range of IPTG (800, 400, 200, 100, 50 µM). Cells were lysed and DnaA protein was detected using Western blot analysis (FtsZ protein was likewise detected and used as a loading control). The amount of DnaA was determined using densitometry; values were normalized to wild-type. Wild-type (HM222), P_spac_-*dnaA* (HM742). (**B**) DNA replication was measured at over a range of DnaA protein levels. Strains were grown as described in (A). Genomic DNA was harvested from cells and marker frequency analysis was determined using qPCR. For each growth media, the *ori:ter* ratios are plotted versus IPTG concentration (error bars indicate the standard deviation of three technical replicates). Representative data are shown from a single experiment; an independently performed replicate of the experiment is shown in Figure S4A. Wild-type (HM222), P_spac_-*dnaA* (HM742). (**C**) Measurement of replication origins number per cell. An array of ∼150 *tetO* sites was inserted near the replication origin and visualized using TetR-YFP. Strains were grown as described in (A), except that overnight cultures were only diluted into a single medium (glycerol + amino acids); AK652 was supplemented with erythromycin and a range of IPTG concentrations. Samples were taken at mid-exponential phase for microscopy and membranes were stained to identify single cells (scale bar  =  5 µm). Histogram colour corresponds to the respective strain/IPTG concentration and the average number of origins per cell is indicated (n> 300). Wild-type (AK647), P_spac_-*dnaA* (AK652). (**D**) To strongly overexpress DnaA the endogenous *dnaA* gene was placed under the control of P_spac_ and an ectopic copy of *dnaA* was integrated at the *amyE* locus under the control of the xylose inducible promoter P_xyl_ (HM745). The strain was grown overnight at 37°C in minimal media supplemented with glycerol, amino acids (20 µg/ml), IPTG (800 µM), and erythromycin. The culture was diluted 1∶100 into media containing IPTG (800 µM), erythromycin, either glycerol minimal media supplemented with a range of xylose (1, 0.5, 0.25, 0.125, 0.063, 0.031, 0.016, 0.008, 0.004, 0%) or LB, and grown at 37°C until an A_600_ of 0.2–0.4. Genomic DNA was harvested from cells and marker frequency analysis was determined using qPCR. For each growth media, the *ori:ter* ratios are plotted versus xylose concentration (error bars indicate the standard deviation of three technical replicates). Representative data are shown from a single experiment; an independently performed replicate of the experiment is shown in Figure S4B. (**E**) HM745 was grown as described in (D) until cultures reached an A_600_ of 0.6–0.9, cells were lysed, and DnaA protein was detected using Western blot analysis (FtsZ protein was likewise detected and used as a loading control). The open arrowhead highlights that overexpressed DnaA ran as a doublet (similar results have been observed for other overexpressed proteins in *B. subtilis*; HM). For each condition the average amount of DnaA (+/− standard deviation) from three biological replicates was determined using densitometry; values were normalized to the cultures without xylose.

To directly test whether the amount of DnaA protein determines the rate of DNA replication initiation, the endogenous *dnaA* gene was placed under the control of an IPTG-inducible promoter (this also alleviated autoregulation of the *dnaA-dnaN* operon [12]). At near wild-type DnaA levels growth rates were normal, *ori:ter* ratios were unchanged, and the distribution of origin regions per cell visualized using a TetR-YFP/*tetO* reporter system was equivalent to wild-type (Figures 2A-C, S4A). In contrast, when the amount of DnaA fell significantly (between ∼50–30% of wild-type, depending upon the media), growth rates slowed, *ori:ter* ratios dropped, DNA replication was inhibited as judged by origin region localization, and cells became elongated (Figures 2A–C, S4A). As noted above *dnaA* is located in an operon upstream of *dnaN* (encoding the sliding clamp component of the replisome) in *B. subtilis*, and Western blot analysis confirmed that the level of DnaN correlated with the level of DnaA (Figure S3B). Depletion of DnaN can cause replication fork stalling and induction of the SOS DNA damage response, which likely contributes to the slow growth and cell elongation phenotypes observed at low IPTG concentrations [12]. However, replication fork stalling would also be expected to cause an increase in the *ori:ter* ratio, suggesting that the observed decreases may be an overestimate of the true initiation frequency. We conclude that wild-type DnaA levels are necessary to achieve the proper frequency of DNA replication initiation at both slow and fast steady-state growth rates.

Only modest overexpression of DnaA could be achieved using the IPTG-inducible promoter (Figure 2A), much lower than the changes in DnaA concentration observed at different nutrient-mediated growth rates (Figure 1C). Therefore, to further increase DnaA protein levels a second copy of the *dnaA* gene was integrated at an ectopic locus under the control of a xylose-inducible promoter (again the endogenous *dnaA-dnaN* operon was expressed using an IPTG-inducible promoter to avoid autorepression). This strain was grown in media that supported a slow growth rate and varying amounts of xylose were added to induce DnaA (>10 fold overexpression was achieved, which was in the range observed for different nutrient-mediated growth rates; Figures 2E, 4D). When DnaA levels were elevated ∼2–4 fold a modest increase in the *ori:ter* ratios was observed, although critically the resulting initiation frequencies remained well below the rate generated in rich media (Figures 2D, S4B). These results indicate that changes in DnaA protein levels are not sufficient to account for nutrient-mediated growth rate regulation of DNA replication initiation in *B. subtilis*.

Surprisingly, further overexpression of DnaA lead to a dramatic decrease in the *ori:ter* ratios. To determine whether this inhibition was specific, DnaA was overexpressed in a strain where *oriC* was inactivated by partial deletion (Δ*oriC*), the endogenous *dnaA-dnaN* operon was expressed using a constitutive promoter to avoid autorepression, and genome replication was driven by a plasmid-derived replication origin (*oriN*; integrated ∼1 kb to the left of *oriC*) that is recognized and activated by its cognate initiator protein (RepN). It is important to note that while initiation at *oriN* does not require either *oriC* or DnaA, the downstream *B. subtilis* initiation proteins DnaD, DnaB and DnaC (helicase) are necessary for *oriN* activity [19]. Therefore, if overexpression of DnaA was either inhibiting the expression of genes required for DNA replication (e.g. – nucleotide biosynthesis [20]) or sequestering essential replication factors, then DNA replication initiation from *oriN* would be expected to decrease. However, overexpression of DnaA in the Δ*oriC oriN^+^* background did not alter *ori:ter* ratios, showing that high overexpression of DnaA specifically inhibits DNA replication initiation at *oriC* (Figure S4C).

### Neither Soj nor YabA nor (p)ppGpp are required for nutrient-mediated growth rate regulation of DNA replication initiation in *B. subtilis*

We hypothesized that nutrient-mediated growth rate control of DNA replication initiation could act via regulation of DnaA activity rather than protein abundance. There are two known trans-acting regulators of *B. subtilis* DnaA during steady-state growth, Soj and YabA. Soj is a dynamic protein that can act as either a negative or a positive regulator of DnaA, depending upon its quaternary state [21]–[23]. YabA is a negative regulator of DnaA that forms a protein bridge between the initiator DnaA and the DNA polymerase sliding clamp processivity factor, DnaN, and is thought to inhibit DNA replication by spatially sequestering DnaA away from the replication origin and by inhibiting DnaA oligomerization [24]–[27]. Interestingly, the number of both proteins per cell was found to positively correlate with growth rate [18].

**Figure 3:**
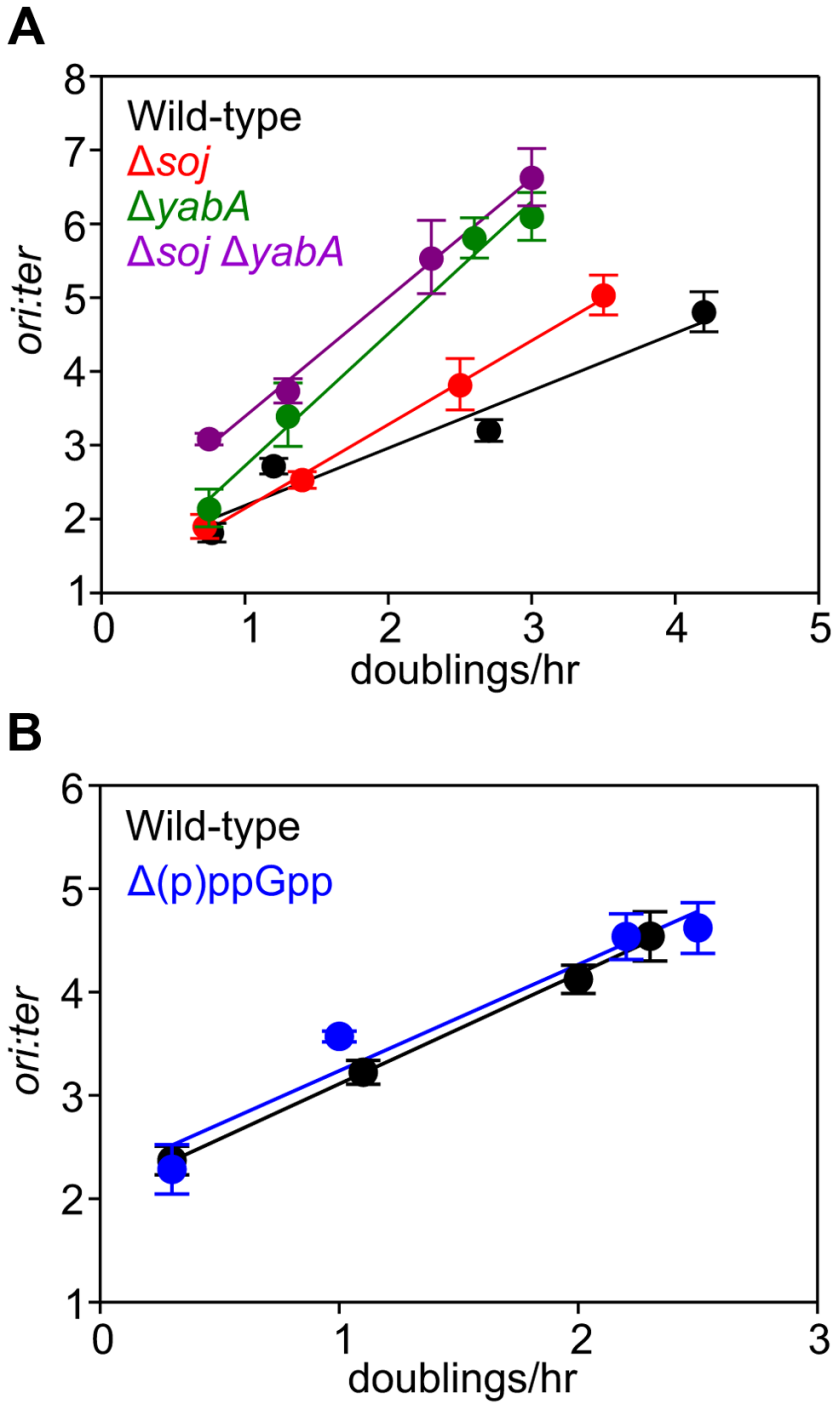
Nutrient-mediated growth rate regulation of DNA replication initiation is independent of Soj, YabA, and (p)ppGpp. (**A**) Growth rate regulation of DNA replication initiation is maintained in either Δ*soj* or Δ*yabA* mutants. Strains were grown overnight at 37°C in minimal media supplemented with succinate and amino acids (20 µg/ml). The culture was diluted 1∶100 into various media (succinate, glycerol, glycerol + amino acids, LB) to generate a range of steady-state growth rates and grown at 37°C until an A_600_ of 0.3–0.4. Genomic DNA was harvested from cells and marker frequency analysis was determined using qPCR. The *ori:ter* ratios are plotted versus growth rate (error bars indicate the standard deviation of three technical replicates). Representative data are shown from a single experiment; an independently performed replicate of the experiment is shown in Figures S5A-B. Wild-type (HM222), Δ*soj* (HM227), Δ*yabA* (HM739), Δ*soj* Δ*yabA* (HM741). (**B**) Growth rate regulation of DNA replication initiation does not require (p)ppGpp. Strains were grown overnight at 37°C in minimal media supplemented with succinate and amino acids (200 µg/ml). The culture was diluted 1∶100 into various media (succinate + amino acids, glycerol + amino acids, LB, PAB) to generate a range of steady-state growth rates and grown at 37°C until an A_600_ of 0.2–0.6. Genomic DNA was harvested from cells and marker frequency analysis was determined using qPCR. The *ori:ter* ratios are plotted versus growth rate (error bars indicate the standard deviation of three technical replicates). Representative data are shown from a single experiment; an independently performed experiment is shown in Figure S5C. Wild-type (HM222), Δ(p)ppGpp (HM1230).

To determine whether either of these regulatory proteins is required for nutrient-mediated growth rate regulation of DNA replication initiation, single knockout mutants were cultured in a range of media and analyzed using marker frequency analysis. It was found that both of the mutant strains retained the ability to coordinate DNA replication initiation with nutrient-mediated changes in growth rate (Figures 3A, S5A). To test whether Soj and YabA acted redundantly to control the nutrient-mediated activity of DnaA, the double mutant was constructed and analysed by marker frequency analysis. Again proper regulation of DNA replication initiation was maintained in the Δ*soj* Δ*yabA* mutant, indicating that neither regulatory protein is required (Figures 3A, S5B). Interestingly, both the single and double mutants displayed a reduced growth rate in rich media, suggesting that the burden of overactive DNA replication initiation may be exacerbated during multifork replication. In the case of the *soj* mutant it is also possible that the slow growth phenotype is related to its role in chromosome origin segregation [28]–[30].

**Figure 4:**
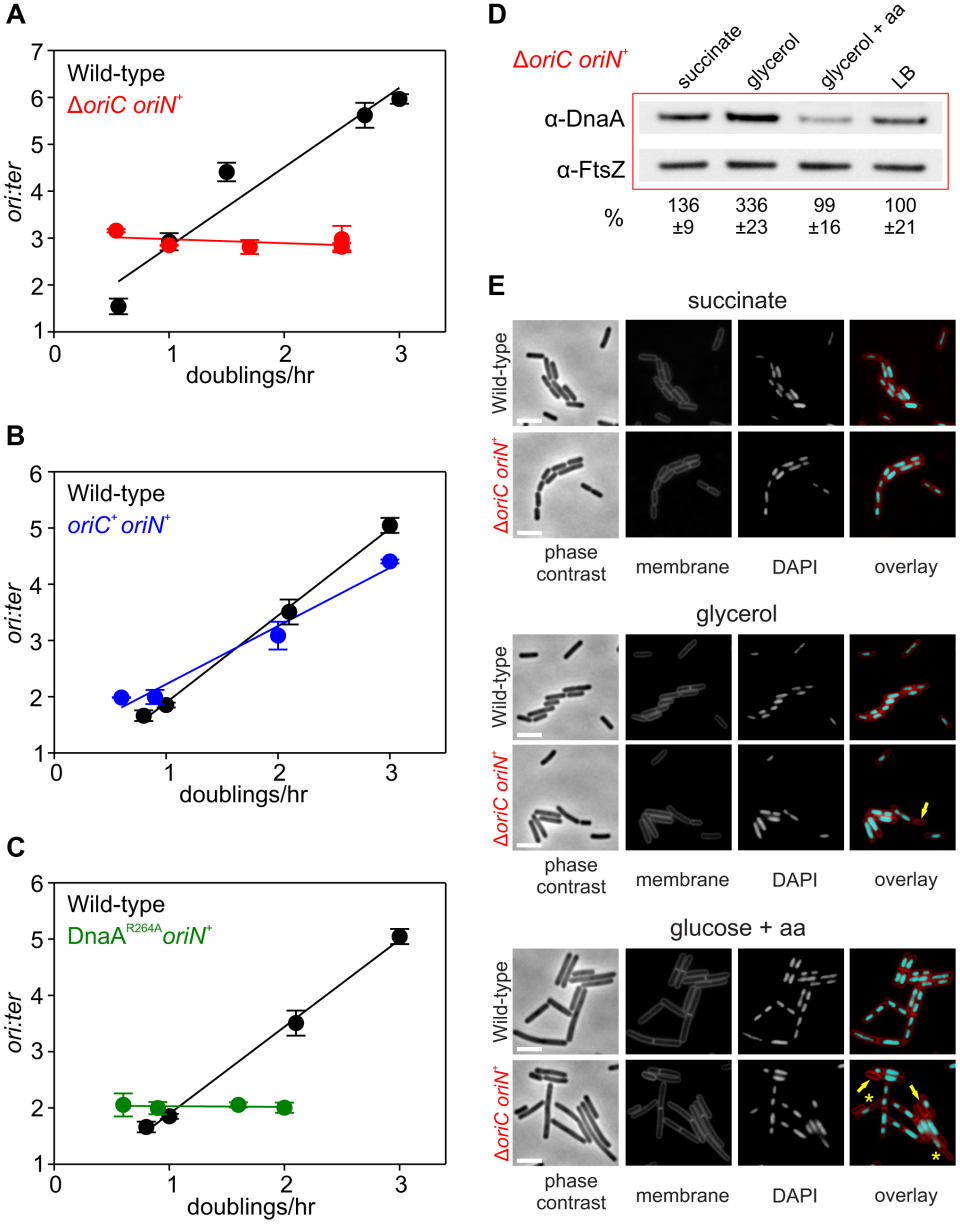
Nutrient-mediated growth rate regulation of DNA replication initiation requires *oriC* and DnaA. (**A**) *oriC* is required for growth rate regulation of DNA replication initiation. Strains were grown overnight at 37°C in minimal media supplemented with succinate and amino acids (20 µg/ml). The culture was diluted 1∶100 into various media (succinate, glycerol, glycerol + amino acids, LB, PAB) to generate a range of steady-state growth rates and grown at 37°C until an A_600_ of 0.3–0.4. Genomic DNA was harvested from cells and marker frequency analysis was determined using qPCR. The *ori:ter* ratios are plotted versus growth rate (error bars indicate the standard deviation of three technical replicates). Representative data are shown from a single experiment; an independently performed replicate of the experiment is shown in Figure S6A. Wild-type (HM222), Δ*oriC oriN^+^* (HM228). (**B**) Integration of *oriN* into the *B. subtilis* chromosome does not eliminate growth rate regulation of DNA replication initiation. Strains were grown as in (A) and the overnight culture was diluted 1∶100 into various media (succinate, glycerol, glycerol + amino acids, LB). Genomic DNA was harvested from cells and marker frequency analysis was determined using qPCR. The *ori:ter* ratios are plotted versus growth rate (error bars indicate the standard deviation of three technical replicates). Representative data are shown from a single experiment; an independently performed replicate of the experiment is shown in Figure S6B. Wild-type (HM715), *oriC* ^+^ *oriN^+^* (HM949). (**C**) DnaA activity is required for growth rate regulation of DNA replication initiation. Strains were grown as in (B). Genomic DNA was harvested from cells and marker frequency analysis was determined using qPCR. The *ori:ter* ratios are plotted versus growth rate (error bars indicate the standard deviation of three technical replicates). Representative data are shown from a single experiment; an independently performed replicate of the experiment is shown in Figure S6C. Wild-type (HM715), DnaA^R264A^ *oriN^+^* (HM1122). (**D**) Measurement of DnaA protein levels at various growth rates in a Δ*oriC oriN^+^* strain (HM950). Cultures were grown as described in (B). Cells were lysed and DnaA protein was detected using Western blot analysis (FtsZ protein was likewise detected and used as a loading control). For each culture media the average amount of DnaA (+/− standard deviation) from at least three biological replicates was determined using densitometry; values were normalized to LB. (**E**) Subcellular localization of DNA over a range of growth rates in the wild-type (HM715) and Δ*oriC oriN^+^* (HM950) strains. Cells were grown as in (B) and the overnight culture was diluted 1∶100 into various media (succinate, glycerol, or glucose + amino acids). Samples were taken at an A_600_ of 0.3–0.5 at which point membranes and DNA were stained. Arrows indicate cells without DNA and asterisks indicate space within the cell that does not contain DNA. Scale bar represents 3 µm.

We also determined whether the alarmone (p)ppGpp, a small molecule induced during nutrient limitation, is required for nutrient-mediated growth rate regulation of DNA replication initiation in *B. subtilis*. Marker frequency analysis was performed on a strain lacking the three known (p)ppGpp synthases (RelA, YwaC, YjbM). It was found that regulation of DNA replication initiation was unaffected by the absence of (p)ppGpp, suggesting that (p)ppGpp is not involved in the regulatory mechanisms coordinating DNA replication with nutrient availability during steady-state cell growth (Figures 3B, S5C).

### DnaA and *oriC* are necessary for nutrient-mediated growth rate regulation of DNA replication initiation in *B. subtilis*

Since both overexpression of DnaA and deletion of DnaA regulatory proteins did not alter nutrient-mediated growth rate regulation of DNA replication initiation, it was unclear whether DnaA was actually a component of this system. To determine whether DnaA activity at *oriC* is required, marker frequency analysis was performed in a strain where *oriC* was inactivated (Δ*oriC*) and DNA replication initiates from the plasmid-derived *oriN*. It was found that *ori:ter* ratios remain constant over a wide range of growth rates in the Δ*oriC* mutant, indicating that nutrient-mediated growth rate regulation of DNA replication was lost (Figures 4A, S6A). Critically, DNA replication initiation from *oriC* is unaffected by the addition of *oriN* (Figures 4B, S6B). This shows that it is the absence of DnaA activity at *oriC*, rather than the presence of *oriN*, which accounts for the loss of nutrient-mediated growth rate regulation in the Δ*oriC* mutant.

However, it could not be concluded whether the Δ*oriC* mutation acted by removing replication origin function or by deleting a site that is required for the nutrient-mediated growth rate regulation. Therefore, a mutation was introduced into *dnaA* that alters the critical “arginine finger” residue (Arg^264^→Ala), thereby disabling DnaA filament assembly and initiation activity (note that a DnaA arginine finger mutant remains competent for DNA binding and ATP binding) [22], [31]. Again the *dnaA^R264A^* mutant strain contains *oriN* in order to maintain viability. Like the Δ*oriC* mutant the DnaA^R264A^ variant also lost growth rate regulation in response to nutrient availability, indicating that DnaA activity at *oriC* is necessary for growth rate regulation in *B. subtilis* (Figures 4C, S6C). Moreover, since the *ori:ter* ratios of the Δ*oriC* and *dnaA^R264A^* mutants remains constant during nutrient-mediated growth rate changes and since DNA replication elongation speed is independent of the nutrient-mediated growth rate [2], [14], the results suggest that within a population of cells the average frequency of DNA replication initiation at *oriN* is independent of the nutrient-mediated growth rate.

Western blot analysis showed that in rich media there was less DnaA and DnaN in the Δ*oriC* strain, whereas conversely there was more DnaA and DnaN in the *dnaA^R264A^* mutant (Figure S3C). The latter result suggests that autoregulation of the *dnaA* promoter requires ATP-dependent filament formation by DnaA, however, the former result was more puzzling. To investigate this further the amount of DnaA in the Δ*oriC* strain was determined over a range of nutrient-mediated growth rates. While the concentration of DnaA was observed to increase as a function of growth rate in the wild-type strain, DnaA levels did not display the same correlation with growth rate in the Δ*oriC* mutant (Figure 4D). This suggests that in the Δ*oriC* mutant nutrient-mediated growth rate-dependent expression of DnaA is lost either because DNA replication initiation from *oriN* is constitutive or because the deletion within *oriC* affects *dnaA* expression (although this region is downstream of the *dnaA* gene).

The apparent decrease in growth rates observed for strains initiating DNA replication solely through *oriN*, particularly in rich media (Figures 4A, 4C, S6A, S6C), is likely due to the formation of cells lacking DNA as a direct consequence of decoupling DNA replication initiation from growth rate (Figure 4E). This result underscores the importance of growth rate regulation of DNA replication initiation to ensure bacterial fitness.

### Slowing growth rate by limiting essential cellular activities inhibits DNA replication

Since nutrient-mediated growth rate regulation of DNA replication initiation did not appear to act through either DnaA protein accumulation or known DnaA regulators, we considered alternative possibilities for how DNA replication could be connected to cell physiology. One hypothesis was that this regulation could be linked to metabolism, either through the amount of a metabolic intermediate or the activity of a critical enzyme. Genetic evidence has suggested a relationship between several glycolytic enzymes and DNA replication in *B. subtilis* and *E. coli* [32], [33], but it has not been established whether these connections act directly at the level of DnaA-dependent initiation. ATP would be another rational candidate since DnaA is an ATP-dependent protein, but it has been found that the concentration of ATP in *B. subtilis* (as well as in *E. coli*) is invariant over a wide range of growth rates (L. Krasny and R. Gourse, personal communication; [34], [35]). Another hypothesis was that this regulation could be linked to the synthesis of an essential cellular complex, such as the ribosome or the cell membrane [36], [37]. In this way a bacterial cell would integrate nutritional information based on the availability of multiple substrates required to construct such macromolecules.

**Figure 5:**
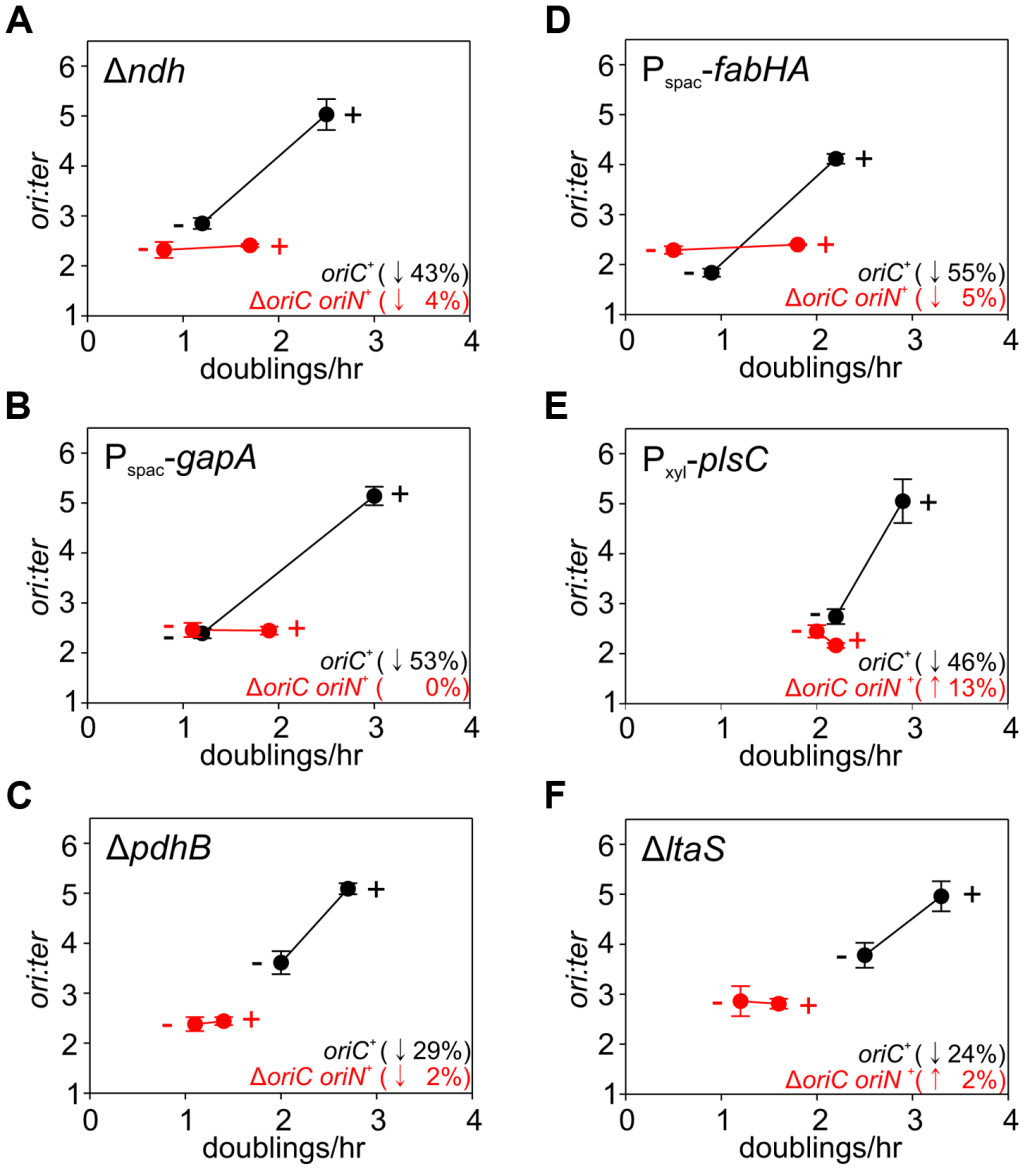
Analysis of *oriC*-dependent growth rate regulation through genetic targeting of essential cellular activities. Strains were grown overnight at 37°C in LB medium; strains harbouring plasmids integrated into the genome by single-crossover were supplemented with appropriate antibiotics and inducer (0.1 mM IPTG or 0.1% xylose). Overnight cultures were diluted 1∶1000 into fresh LB medium and grown at 37°C until they reached an A_600_ of 0.3–0.5; strains harbouring plasmids integrated by single-crossover were supplemented with appropriate antibiotics either without or with the appropriate inducer (1 mM IPTG or 1% xylose). For datapoints “+” indicates the presence of either the wild-type gene (when comparing with knockout mutants) or the inducer; “−” indicates the absence of either the gene (when comparing with wild-type) or the inducer. Genomic DNA was harvested from cells and marker frequency analysis was determined using qPCR. The *ori:ter* ratios are plotted versus growth rate and the percentage change in the *ori:ter* ratios comparing each deletion/depletion is indicated (error bars indicate the standard deviation of three technical replicates). Representative data are shown from a single experiment; an independently performed replicate of the experiment is shown in Figure S7. (**A**) Wild-type (HM715), Δ*ndh* (HM1318), Δ*oriC oriN^+^* (HM957), Δ*ndh* Δ*oriC oriN^+^* (HM1319); (**B**) P_spac_-*gapA* (HM1208), P_spac_-*gapA* Δ*oriC oriN^+^* (HM1221); (**C**) Cultures were supplemented with 0.2% sodium acetate. Wild-type (HM715), Δ*pdhB* (HM1248), Δ*oriC oriN^+^* (HM950), Δ*pdhB* Δ*oriC oriN^+^* (HM1266); (**D**) P_spac_-*fabHA* (HM964), P_spac_-*fabHA* Δ*oriC oriN^+^* (HM966); (**E**) P_xyl_-*plsC* (HM1080), P_xyl_-*plsC* Δ*oriC oriN^+^* (HM1086); (**F**) Wild-type (HM715), Δ*ltaS* (HM1168), Δ*oriC oriN^+^* (HM957), Δ*ltaS* Δ*oriC oriN^+^* (HM1244).

**Figure 6:**
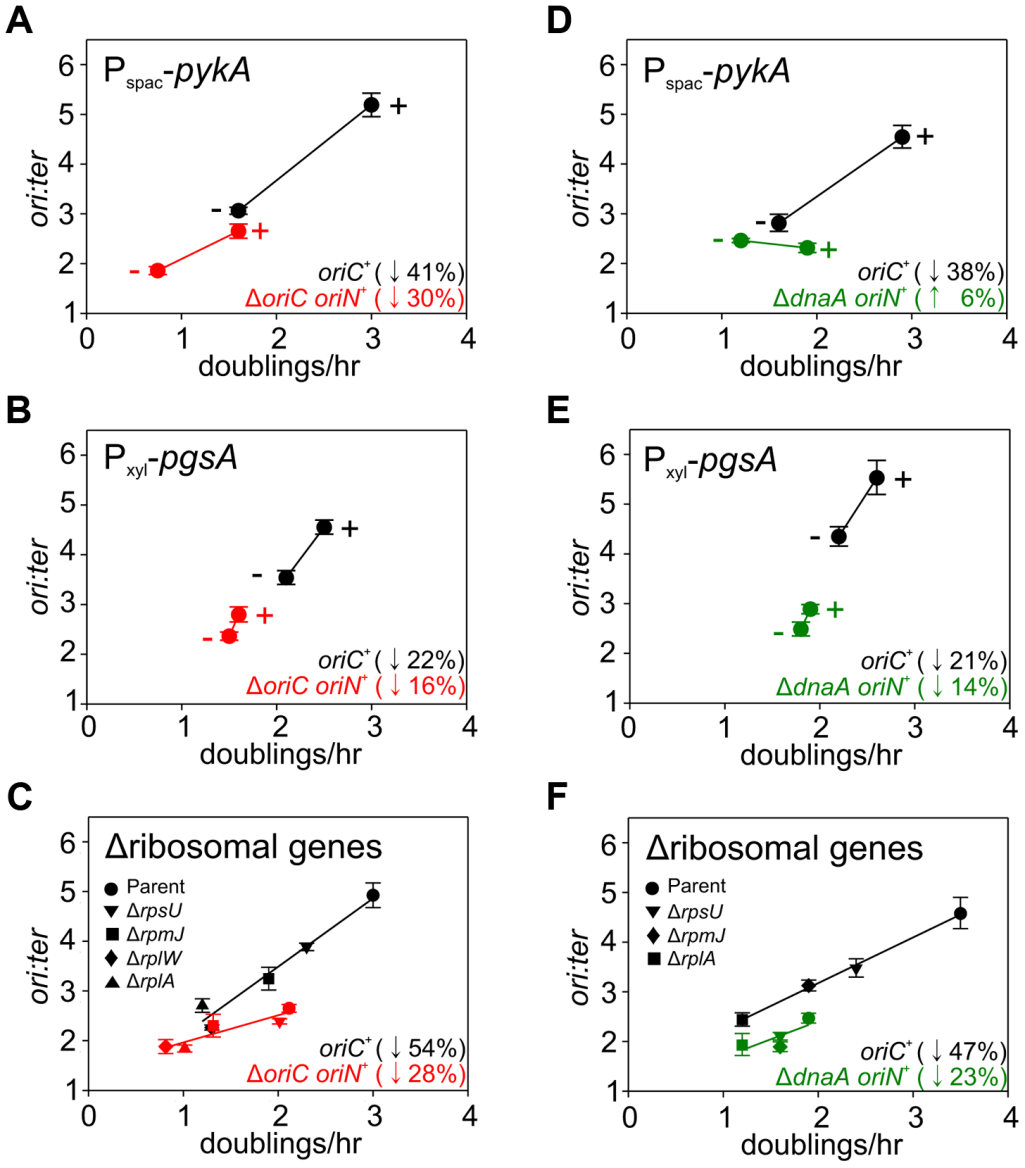
Analysis of *oriC*-independent growth rate regulation through genetic targeting of essential cellular activities. Strains were grown and data presented as described for Figure 5, except that the depletion of PgsA required supplementation with 1 mM IPTG to overexpress the xylose repressor. The *ori:ter* ratios are plotted versus growth rate and the percentage change in the *ori:ter* ratios comparing each deletion/depletion is indicated (error bars indicate the standard deviation of three technical replicates). Representative data are shown from a single experiment; an independently performed replicate of the experiment is shown in Figure S8. (**A**) P_spac_-*pykA* (HM1176), P_spac_-*pykA* Δ*oriC oriN^+^* (HM1186); (**B**) P_xyl_-*pgsA* (HM1365), P_xyl_-*pgsA* Δ*oriC oriN^+^* (HM1374); (**C**) Wild-type (HM715), Δ*rpsU* (HM1150), Δ*rplA* (HM1151), Δ*rplW* (HM1152), Δ*rpmJ* (HM1154), Δ*oriC oriN^+^* (HM950), Δ*rpsU* Δ*oriC oriN^+^* (HM1156), Δ*rplA* Δ*oriC oriN^+^* (HM1157), Δ*rplW* Δ*oriC oriN^+^* (HM1158), Δ*rpmJ* Δ*oriC oriN^+^* (HM1160). (**D**) P_spac_-*pykA* (HM1176), P_spac_-*pykA* Δ*dnaA oriN^+^* (HM1425); (**E**) P_xyl_-*pgsA* (HM1365), P_xyl_-*pgsA* Δ*dnaA oriN^+^* (HM1433); (**F**) Wild-type (HM715), Δ*rpsU* (HM1150), Δ*rplA* (HM1151), Δ*rpmJ* (HM1154), Δ*dnaA oriN^+^* (HM1423), Δ*rpsU* Δ*dnaA oriN^+^* (HM1429), Δ*rplA* Δ*dnaA oriN^+^* (HM1430), Δ*rpmJ* Δ*dnaA oriN^+^* (HM1432).

In order to identify possible routes through which nutrient availability could impact DNA replication we analyzed a range of genetically altered strains, targeting respiration, central carbon metabolism, protein synthesis, fatty acid synthesis, and phospholipid synthesis (Table 1), that all manifest decreased steady-state growth rates in rich complex media. Genes were either disrupted by antibiotic cassettes or depleted using regulated expression systems; importantly, depletion of essential genes was not lethal under the experimental conditions used. Knock-out strains were compared to wild-type while depletion strains were analyzed without and with inducer (indicated in Figures 5, 6, S7, S8 with “-” and “+”, respectively). DNA replication was measured using marker frequency analysis. Strikingly, in all of the strains examined the *ori:ter* ratio decreased to match the slower growth rates caused by gene disruption/depletion (Figures 5–6, S7–S8; black symbols).

**Table 1:** **Genes manipulated to limit essential cellular processes.**

Gene	Protein	Cellular Activity
*ndh*	NADH dehydrogenase	respiration
*gapA*	glyceraldehyde-3-P dehydrogenase	central carbon metabolism
*pykA*	pyruvate kinase	central carbon metabolism
*pdhB*	pyruvate dehydrogenase (E1 β subunit)	central carbon metabolism
*fabHA*	β-ketoacyl-acyl carrier protein synthase III	fatty acid synthesis
*ltaS*	lipoteichoic acid synthase	lipoteichoic acid synthesis
*plsC*	acyl-ACP:1-acylglycerolphosphate acyltransferase	phospholipid synthesis
*pgsA*	phosphatidylglycerophosphate synthase	phospholipid synthesis
*rpsU*	ribosomal protein S21	protein synthesis
*rplA*	ribosomal protein L1	protein synthesis
*rplW*	ribosomal protein L23	protein synthesis
*rpmJ*	ribosomal protein L36	protein synthesis

### Evidence for an *oriC*-independent response to changes in growth rate

The uniform response of DNA replication in slow growing mutants suggested that a single mechanism might account for this regulation, in accord with nutrient-mediated regulation of DNA replication initiation (Figures 4, S6). To examine this hypothesis the deletion and depletion strains were crossed into the Δ*oriC* strain that initiates DNA replication using *oriN*. For several mutants (*ndh*, *gapA*, *pdhB*, *fabHA*, *plsC* and *ltaS*) the *ori:ter* ratio was not significantly affected (≤1/10 of the percentage decrease observed for *oriC^+^*), suggesting that the regulatory signal specifically targeted DNA replication initiation at *oriC* (Figures 5, S7; red symbols). However, there were a number of mutants (*pykA, pgsA*, and multiple ribosomal protein genes) that produced a marked decrease in the *ori:ter* ratio of the Δ*oriC* strain (≥1/2 of the percentage decrease observed for *oriC^+^*), suggesting that in these cases DNA replication was being regulated through an *oriC*-independent mechanism (Figures 6A–C, S8A-C; red symbols). Interestingly, in some cases manipulation of different genes within a single biological pathway (e.g. – carbon metabolism or phospholipid synthesis) resulted in the regulation of DNA replication through the different regulatory systems.

### Evidence for a DnaA-independent response to changes in growth rate

Since the Δ*oriC oriN* ^+^ strain does not require DnaA activity to initiate DNA replication, it suggested that the observed *oriC*-independent growth rate regulation might be DnaA-independent. To address this possibility the *oriC*-independent mutants (Figures 6A–C, S8A–C) were crossed into a Δ*dnaA* strain that initiates DNA replication using *oriN* (Figure S3C). When PykA was depleted in the Δ*dnaA* mutant the *ori:ter* ratio no longer decreased, indicating that DnaA was required for this response, although apparently not for its role in origin recognition and DNA unwinding (Figures 6D, S8D; green symbols). In contrast, when either ribosomal genes were deleted or PgsA was depleted in the Δ*dnaA* mutant the *ori:ter* ratios did decrease, suggesting that DnaA-independent mechanisms act under these conditions (Figures 6E–F, S8E–F; green symbols). Taken together, the genetic analysis reveals that in *B. subtilis* there is likely more than one regulatory system linking DNA replication with cell growth.

### Slowing growth rate by targeting essential cellular activities with small molecules inhibits DNA replication initiation

**Figure 7:**
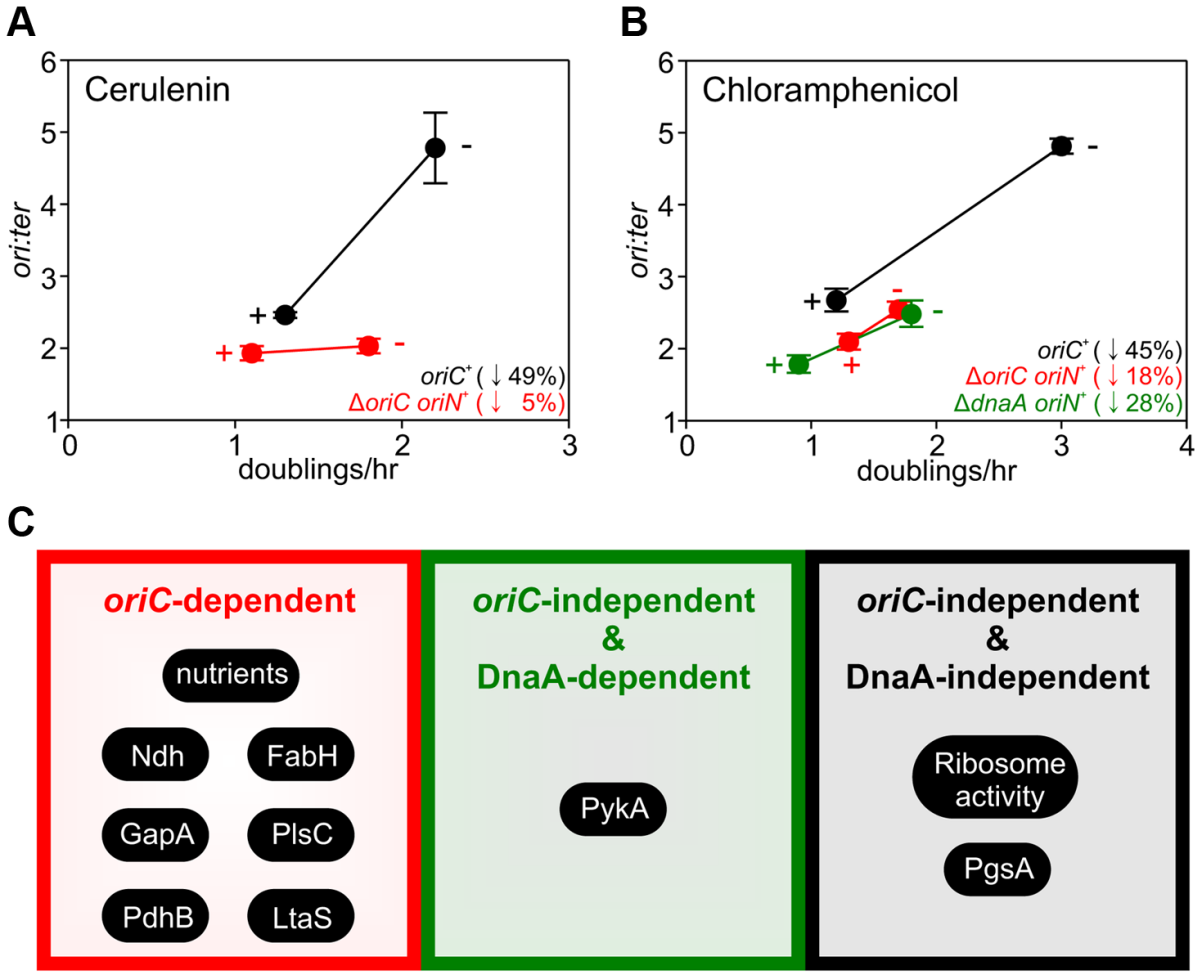
Analysis of *oriC*-dependent and *oriC*-independent growth rate regulation through small molecule targeting of fatty acid synthesis and protein synthesis. Strains were grown overnight at 37°C in LB medium. Overnight cultures were diluted 1∶1000 into fresh LB medium either without or with antibiotics (2 µg/ml cerulenin (**A**), 1 µg/ml chloramphenicol (**B**)) and grown at 37°C until they reached an A_600_ of 0.3–0.5. For datapoints “+” indicates the presence of the small molecule inhibitor and “−” indicates the absence. Genomic DNA was harvested from cells and marker frequency analysis was determined using qPCR. The *ori:ter* ratios are plotted versus growth rate and the percentage change in the *ori:ter* ratios comparing each deletion/depletion is indicated (error bars indicate the standard deviation of three technical replicates). Representative data are shown from a single experiment; an independently performed replicate of the experiment is shown in Figure S9. Wild-type (HM715), Δ*oriC oriN^+^* (HM950), Δ*dnaA oriN^+^* (HM1423). (**C**) Summary of growth rate control systems affecting DNA replication described in this report.

The importance of growth rate regulation of DNA replication in response to nutrient availability is self-evident, but the biological relevance of growth rate regulation of DNA replication in response to genetic manipulations is less clear. To address this issue we evaluated the response of DNA replication to sublethal concentrations of antibiotics that produced slow steady-state growth rates. We chose small molecules that inhibit either fatty acid synthesis (cerulenin) or protein synthesis (chloramphenicol) because our genetic analyses indicated that the former regulated DNA replication through *oriC* while the latter acted independently of both *oriC* and DnaA. Incubation with either antibiotic caused a decrease in the *ori:ter* ratios in the wild-type strain, showing that growth rate regulation of DNA replication in response to genetic perturbations of essential cellular activities is physiologically relevant (Figures 7A–B, S9A–B; black symbols).

To further assess whether changes in DNA replication caused by these small molecules reflected the results using genetic approaches, the Δ*oriC oriN^+^* strain was analyzed. Only chloramphenicol elicited a significant decrease in the *ori:ter* ratios in the Δ*oriC* strain (Figures 7A–B, S9A–B; red symbols). Finally, the Δ*dnaA oriN^+^* strain was analyzed in the presence of chloramphenicol and again the *ori:ter* ratio decreased (Figures 7B, S9B). This result is fully consistent with the data from ribosomal gene deletions and indicates that regulation of DNA replication in response to perturbed ribosome activity is DnaA-independent.

## Discussion

We have found that nutrient-mediated growth rate regulation of DNA replication initiation in *B. subtilis* requires both DnaA and *oriC*. To our knowledge this is the first time that a specific DNA replication initiation protein has been shown to play an essential role in this regulatory system, and because DnaA is the earliest acting initiation factor we propose that DnaA is the target for the nutrient-mediated growth rate regulatory system. Critically however, we show that changes in DnaA protein level are not sufficient to account for nutrient-mediated growth rate regulation of DNA replication initiation in *B. subtilis*. This is in contrast to the generally accepted mechanism for control of bacterial DNA replication initiation based on work using *E. coli* [8], [9].

*B. subtilis* contains a bipartite origin that flanks the *dnaA* gene (*incA* and *incB* regions containing the *dnaA* promoter lie 1.3 kb upstream of the *incC* region which contains the DNA unwinding element) [38]. When the expression of the *dnaA-dnaN* operon was placed under the control of the inducible P_spac_ promoter in order to test the effect of DnaA overexpression on DNA replication initiation, a ∼9kb plasmid was recombined upstream of *dnaA* by single cross-over. Therefore, integration of this vector resulted in the considerable displacement of the two origin regions from one another without any significant consequence. It will be extremely interesting to determine the maximum and minimum distances that the *inc* regions can be moved, as well as ascertaining the role of the upstream region in DNA replication initiation.

We have shown that neither of the known DnaA regulatory proteins present during vegetative growth, Soj and YabA, are required for nutrient-mediated growth rate regulation of DNA replication initiation. We have also determined that the alarmone (p)ppGpp is not required for this regulation, consistent with a previous report that induction of the stringent response inhibits DNA replication elongation but not initiation in *B. subtilis* [39], [40]. This result marks an apparent distinction between the role of (p)ppGpp in *B. subtilis* and in proteobacteria such as *E. coli* and *Caulobacter crescentus* where (p)ppGpp has been shown to regulate DNA replication initiation [41], [42].

The use of genetic manipulations and small molecule inhibitors presented here reinforce and extend previously observed connections for bacterial DNA replication with central carbon metabolism [32], [33] and with phospholipid synthesis [43], [44]. In addition our work identifies new links for *B. subtilis* DNA replication with respiration, fatty acid synthesis, lipoteichoic acid synthesis, and ribosome biosynthesis. The results indicate that growth rate regulation of DNA replication in *B. subtilis* can be controlled through either *oriC*-dependent, *oriC*-independent/DnaA-dependent, or *oriC*-independent/DnaA-independent mechanisms (summarized in Figure 7C). Based on these novel findings we propose that multiple systems coordinate DNA replication with bacterial cell growth, with distinct regulators responding to diverse physiological and chemical changes. This model deviates from the long-standing concept of a single universal cellular property utilized to link bacterial DNA replication with cell growth [4].

Since nutrient-mediated growth rate regulation of DNA replication initiation requires DnaA activity at *oriC*, this suggests that factors affecting DNA synthesis through an *oriC*-independent mechanism (PykA, PgsA, and ribosomal proteins) are unlikely to be responsible for the nutrient sensing system. We suspect that nutrient-mediated regulation may be influenced by more than one control system, thereby forming a robust network capable of integrating information from multiple metabolic and cellular sources.

We note that for deletion/depletion mutants regulating DNA replication through *oriC*-dependent and *oriC*-independent/DnaA-dependent mechanisms, *ori:ter* ratios either remained constant or slightly increased in the Δ*oriC* and Δ*dnaA* strains, respectively (Figures 5, 6D, S7, S8D). Because the average initiation frequency of *oriN* appears to be growth rate independent (Figures 4A, 4C, S6A, S6C), the measured *ori:ter* ratios of these strains indicates that replication elongation speeds are either not being affected or are slightly decreasing. Therefore, for both *oriC*-dependent and *oriC*-independent/DnaA-dependent regulatory mechanisms, the observed decrease in *ori:ter* ratios in the *oriC^+^* strain most likely reflects inhibition of DNA replication initiation. In contrast, for the *oriC*-independent/DnaA-independent mutants where the *ori:ter* ratio was decreased when DNA replication initiated from *oriN*, this difference could be due to a change in DNA replication elongation (although this would mean that the elongation speed was increased).

Our current aim is to determine the molecular mechanisms underlying each system that coordinates DNA replication with cell growth. We hypothesize that the *oriC*-dependent regulatory system targets DnaA activity at *oriC*. We speculate that the *oriC*-independent/DnaA-dependent regulatory system could influence DNA replication initiation by affecting the abundance or activity of a downstream replication initiation factor. For example, DnaA is also a transcription factor that is thought to directly regulate the expression of>50 genes, including *dnaB* [20], [45]. Alternatively, DnaA could act by titrating initiation factors away from *oriN*. Lastly, in the case of the *oriC*-independent/DnaA-independent regulatory system it needs to be established whether DNA replication is affected at the step of initiation or elongation, after which the role of appropriate candidate proteins can be investigated.

## Materials and Methods

### Strains and plasmids

Strains are listed in Table S1. Plasmids are listed in Table S2 and Table S3.

### Media and chemicals

Nutrient agar (NA; Oxoid) was used for routine selection and maintenance of both *B. subtilis* and *E. coli* strains. For experiments in *B. subtilis* cells were grown using a range of media (using the following concentrations unless otherwise noted): Luria-Bertani (LB) medium, Antibiotic 3 (PAB) medium, Brain-Heart Infusion (Bacto), or defined minimal medium base (Spizizen minimal salts supplemented with Fe-NH_4_-citrate (1 µg/ml), MgSO_4_ (6 mM), CaCl_2_ (100 µM), MnSO_4_ (130 µM), ZnCl_2_ (1 µM), thiamine (2 µM)) supplemented with casein hydrolysate (200 µg/ml) and/or various carbon sources (succinate (1%), glycerol (0.5%), glucose (0.5%)). Supplements were added as required: tryptophan (20 µg/ml), phenylalanine (40 µg/ml), chloramphenicol (5 µg/ml), erythromycin (1 µg/ml), kanamycin (2 µg/ml), spectinomycin (50 µg/ml), tetracycline (10 µg/ml), zeocin (10 µg/ml). Unless otherwise stated all chemicals and reagents were obtained from Sigma-Aldrich.

### Marker frequency analysis

Sodium azide (0.5%; Sigma) was added to exponentially growing cells to prevent further metabolism. Chromosomal DNA was isolated using a DNeasy Blood and Tissue Kit (Qiagen). The DNA replication origin (*oriC*) region was amplified using primers 5′-GAATTCCTTCAGGCCATTGA-3′ and 5′-GATTTCTGGCGAATTGGAAG-3′; the DNA replication terminus (*ter*) region was amplified using primers 5′-TCCATATCCTCGCTCCTACG-3′ and 5′-ATTCTGCTGATGTGCAATGG-3′. Either Rotor-Gene SYBR Green (Qiagen) or GoTaq (Promega) qPCR mix was used for PCR reactions. Q-PCR was performed in a Rotor-Gene Q Instrument (Qiagen). By use of crossing points (C_T_) and PCR efficiency a relative quantification analysis (ΔΔC_T_) was performed using Rotor-Gene Software version 2.0.2 (Qiagen) to determine the origin:terminus (*ori:ter*) ratio of each sample. These results were normalized to the *ori:ter* ratio of a DNA sample from *B. subtilis* spores which only contain one chromosome and thus have an *ori/ter* ratio of 1.

### Microscopy

To visualize cells during exponential growth starter cultures were grown overnight and then diluted 1∶100 into fresh medium and allowed to achieve at least three doublings before observation. Cells were mounted on ∼1.2% agar pads (0.25× minimal medium base) and a 0.13–0.17 mm glass coverslip (VWR) was placed on top. To visualize individual cells the cell membrane was stained with either 2 µg/ml Nile Red (Sigma) or 0.4 µg/ml FM5-95 (Molecular Probes). To visualize nucleoids DNA was stained with 2 µg/ml 4′-6-diamidino-2-phenylindole (DAPI) (Sigma). Microscopy was performed on an inverted epifluorescence microscope (Nikon Ti) fitted with a Plan-Apochromat objective (Nikon DM 100x/1.40 Oil Ph3). Light was transmitted from a 300 Watt xenon arc-lamp through a liquid light guide (Sutter Instruments) and images were collected using a CoolSnap HQ^2^ cooled CCD camera (Photometrics). All filters were Modified Magnetron ET Sets from Chroma and details are available upon request. Digital images were acquired and analysed using METAMORPH software (version V.6.2r6). Analysis was performed using ImageJ software: foci counting utilized the particle analysis plugin; cell lengths and widths were measured using the ObjectJ plugin.

### Western blot analysis

Proteins were separated by electrophoresis using a NuPAGE 4-12% Bis-Tris gradient gel run in MES buffer (Life Technologies) and transferred to a Hybond-P PVDF membrane (GE Healthcare) using a semi-dry apparatus (Hoefer Scientific Instruments). Proteins of interest were probed with polyclonal primary antibodies and then detected with an anti-rabbit horseradish peroxidase-linked secondary antibody using an ImageQuant LAS 4000 mini digital imaging system (GE Healthcare). Quantification was determined by densitometry using Image J software. Figure S3A shows that detection of DnaA, FtsZ and DnaN was within a linear range.

## Supporting Information

Figure S1 Culturing *B. subtilis* at different temperatures generates a range of steady-state growth rates but does not affect the frequency of DNA replication initiation. A wild-type strain (HM715) was grown overnight at 23°C in LB. The culture was diluted 1:100 into LB and incubated at different temperatures to generate a range of steady-state growth rates until an A_600_ of 0.2-0.3. Genomic DNA was harvested from cells and marker frequency analysis was determined using qPCR. The *ori:ter* ratios are plotted versus growth rate (error bars indicate the standard deviation of three technical replicates). Representative data are shown from a single experiment; an independently performed replicate of the experiment is shown in Figure 1B.
(PDF)

Figure S2 Cell measurements as a function of nutrient-mediated growth rate. **(A)** Measurement of replication origins per cell. An array of ∼25 *tetO* sites was inserted near the replication origin and was visualized using TetR-GFP. Strain AK47 was grown overnight at 37°C in minimal media supplemented with succinate (2%), amino acids (0.2%), spectinomycin (50 µg/ml) and erythromycin (1 µg/ml). Cultures were washed twice and diluted 1∶100 into various chemically defined media supplemented with either succinate (2%), glucose (1.5%), or glucose (1.5%) with amino acids (200 µg/ml) and grown at 37°C until they reached an A_600_ of 0.3–0.5. Samples were taken for microscopy and membranes were stained. Scale bar represents 3 µm. **(B)** Quantification of the number of origins per cell at different growth rates. The average number of origins per cell is indicated above each histogram. **(C,E)** Cell lengths were grouped according to the number of origins, measurements were binned in 0.5 µm steps, and data plotted as a percentage within each population. **(D,F)** The average cell lengths and widths (+/− standard deviation) were grouped according to the number of origins per cell.
(PDF)

Figure S3 Western blot analysis of wild-type, *oriN*, and P_spac_-*dnaA-dnaN* strains. Strains were grown overnight at 37°C in LB medium. Overnight cultures were diluted 1∶1000 into fresh LB medium and grown at 37°C until an A_600_ of 0.5-0.7 was attained. Cells were lysed and proteins were detected using Western blot analysis. **(A)** A two-fold dilution series of a cell lysate was used to determine the linear range for each antibody. **(B)** The endogenous *dnaA-dnaN* operon was placed under the control of the IPTG-inducible promoter P_spac_ to generate a range of expression levels. HM742 was supplemented with IPTG (400 µM) and erythromycin. The cultures were diluted 1∶100 into LB and grown at 37°C until an A_600_ of 0.5–0.6; HM742 was supplemented with erythromycin and a range of IPTG (800, 400, 200, 100, 50 µM). Cells were lysed and DnaN protein was detected using Western blot analysis (FtsZ protein was likewise detected and used as a loading control). The amount of DnaN was determined using densitometry; values were normalized to wild-type. Wild-type (HM222), P_spac_-*dnaA-dnaN* (HM742). **(C)** Analysis of *oriN* strains. Wild-type (HM715), *oriC^+^ oriN^+^* (HM949), Δ*oriC oriN^+^* (HM957), Δ*dnaA oriN^+^* (HM1423), *dnaA^R264A^ oriN^+^* (HM1122).
(PDF)

Figure S4 Changes in DnaA protein level are not sufficient to account for nutrient-mediated growth rate regulation of DNA replication initiation in *B. subtilis*. **(A)** The endogenous *dnaA* gene was placed under the control of the IPTG-inducible promoter P_spac_ to generate a range of DnaA protein levels. Strains were grown overnight at 37°C in minimal media supplemented with succinate and amino acids (20 µg/ml); IPTG (400 µM) and erythromycin was added to HM742. The cultures were diluted 1∶100 into various media (glycerol, glycerol + amino acids, LB) to generate a range of steady-state growth rates and grown at 37°C until an A_600_ of 0.5–0.6; in each medium HM742 was supplemented with erythromycin and a range of IPTG (800, 400, 200, 100, 50 µM). Genomic DNA was harvested from cells and marker frequency analysis was determined using qPCR. For each growth media, the *ori:ter* ratios are plotted versus IPTG concentration (error bars indicate the standard deviation of three technical replicates). Representative data are shown from a single experiment; an independently performed replicate of the experiment is shown in Figure 2B. Wild-type (HM222), P_spac_-*dnaA* (HM742). **(B)** To strongly overexpress DnaA the endogenous *dnaA* gene was placed under the control of P_spac_ and an ectopic copy of *dnaA* was integrated at the *amyE* locus under the control of the xylose inducible promoter P_xyl_ (HM745). The strain was grown overnight at 37°C in minimal media supplemented with glycerol, amino acids (20 µg/ml), IPTG (800 µM), and erythromycin. The culture was diluted 1∶100 into media containing IPTG (800 µM), erythromycin, either glycerol minimal media supplemented with a range of xylose (1, 0.5, 0.25, 0.125, 0.063, 0.031, 0.016, 0.008, 0.004, 0%) or LB, and grown at 37°C until an A_600_ of 0.2–0.4. Genomic DNA was harvested from cells and marker frequency analysis was determined using qPCR. For each growth media, the *ori:ter* ratios are plotted versus xylose concentration (error bars indicate the standard deviation of three technical replicates). Representative data are shown from a single experiment; an independently performed replicate of the experiment is shown in Figure 2D. **(C)** To determine whether overexpression of DnaA specifically inhibits DNA replication initiation from *oriC*, the endogenous *dnaA* gene was placed under the control of P_spac_, an ectopic copy of *dnaA* was integrated at the *amyE* locus under the control of the xylose inducible promoter P_xyl_, *oriC* was inactivated by partial deletion (Δ*incAB*), and DNA replication was driven by *oriN* (HM1467). The strain was grown overnight at 37°C in minimal media supplemented with glycerol, amino acids (20 µg/ml), IPTG (800 µM), and erythromycin. The culture was diluted 1∶100 into glycerol minimal media containing IPTG (800 µM), erythromycin, supplemented with a range of xylose (1, 0.5, 0.25, 0.125, 0.063, 0.031, 0.016, 0.008, 0.004, 0%), and grown at 37°C until an A_600_ of 0.2–0.4. Genomic DNA was harvested from cells and marker frequency analysis was determined using qPCR. The *ori:ter* ratios are plotted versus xylose concentration (error bars indicate the standard deviation of three technical replicates).
(PDF)

Figure S5 Nutrient-mediated growth rate regulation of DNA replication initiation is independent of Soj, YabA, and (p)ppGpp. **(A)** Growth rate regulation of DNA replication initiation is maintained in either Δsoj or ΔyabA mutants. Strains were grown overnight at 37°C in minimal media supplemented with succinate and amino acids (20 µg/ml). The culture was diluted 1∶100 into various media (succinate, glycerol, glycerol + amino acids, LB) to generate a range of steady-state growth rates and incubated at 37°C until an A_600_ of 0.3–0.4. Genomic DNA was harvested from cells and marker frequency analysis was determined using qPCR. The *ori:ter* ratios are plotted versus growth rate (error bars indicate the standard deviation of three technical replicates). Representative data are shown from a single experiment; an independently performed replicate of the experiment is shown in Figure 3A. Wild-type (HM222), Δ*soj* (HM227), Δ*yabA* (HM739). **(B)** Growth rate regulation of DNA replication initiation is maintained in a Δ*soj* Δ*yabA* double mutant. Cells were grown as in (A). Genomic DNA was harvested from cells and marker frequency analysis was determined using qPCR. The *ori:ter* ratios are plotted versus growth rate (error bars indicate the standard deviation of three technical replicates). Representative data are shown from a single experiment; an independently performed replicate of the experiment is shown in Figure 3A. Wild-type (HM222), Δ*soj* Δ*yabA* (HM741). **(C)** Growth rate regulation of DNA replication initiation does not require (p)ppGpp. Strains were grown overnight at 37°C in minimal media supplemented with succinate and amino acids (200 µg/ml). The culture was diluted 1∶100 into various media (succinate + amino acids, glycerol + amino acids, LB) to generate a range of steady-state growth rates and incubated at 37°C until an A_600_ of 0.2–0.6. Genomic DNA was harvested from cells and marker frequency analysis was determined using qPCR. The *ori:ter* ratios are plotted versus growth rate (error bars indicate the standard deviation of three technical replicates). Representative data are shown from a single experiment; an independently performed experiment is shown in Figure 3B. Wild-type (PY79), Δ(p)ppGpp (bSS186).
(PDF)

Figure S6 Nutrient-mediated growth rate regulation of DNA replication initiation requires *oriC* and DnaA. **(A)** *oriC* is required for growth rate regulation of DNA replication initiation. Strains were grown overnight at 37°C in minimal media supplemented with succinate and amino acids (20 µg/ml). The culture was diluted 1∶100 into various media (succinate, glycerol, glycerol + amino acids, LB) to generate a range of steady-state growth rates and incubated at 37°C until an A_600_ of 0.3–0.4. Genomic DNA was harvested from cells and marker frequency analysis was determined using qPCR. The *ori:ter* ratios are plotted versus growth rate (error bars indicate the standard deviation of three technical replicates). Representative data are shown from a single experiment; an independently performed replicate of the experiment is shown in Figure 4A. Wild-type (HM715), Δ*oriC oriN^+^* (HM950). **(B)** Integration of *oriN* into the *B. subtilis* chromosome does not eliminate growth rate regulation of DNA replication initiation. Strains were grown as in (A). Genomic DNA was harvested from cells and marker frequency analysis was determined using qPCR. The *ori:ter* ratios are plotted versus growth rate (error bars indicate the standard deviation of three technical replicates). Representative data are shown from a single experiment; an independently performed replicate of the experiment is shown in Figure 4B. Wild-type (HM715), *oriC* ^+^ *oriN^+^* (HM949). **(C)** DnaA activity is required for growth rate regulation of DNA replication initiation. Strains were grown as in (B). Genomic DNA was harvested from cells and marker frequency analysis was determined using qPCR. The *ori:ter* ratios are plotted versus growth rate (error bars indicate the standard deviation of three technical replicates). Representative data are shown from a single experiment; an independently performed replicate of the experiment is shown in Figure 4C. Wild-type (HM715), DnaA^R264A^ *oriN^+^* (HM1122).
(PDF)

Figure S7 Analysis of *oriC*-dependent growth rate regulation through genetic targeting of essential cellular activities. Strains were grown overnight at 37°C in LB medium; strains harbouring plasmids integrated into the genome by single-crossover were supplemented with appropriate antibiotics and inducer (0.1 mM IPTG or 0.1% xylose). Overnight cultures were diluted 1∶1000 into fresh LB medium and grown at 37°C until they reached an A_600_ of 0.3–0.5; strains harbouring plasmids integrated by single-crossover were supplemented with appropriate antibiotics either without or with the appropriate inducer (1 mM IPTG or 1% xylose). For datapoints “+” indicates the presence of either the wild-type gene (when comparing with knockout mutants) or the inducer; “−” indicates the absence of either the gene (when comparing with wild-type) or the inducer. Genomic DNA was harvested from cells and marker frequency analysis was determined using qPCR. The *ori:ter* ratios are plotted versus growth rate and the percentage change in the *ori:ter* ratios comparing each deletion/depletion is indicated (error bars indicate the standard deviation of three technical replicates). Representative data are shown from a single experiment; an independently performed replicate of the experiment is shown in Figure 5. **(A)** Wild-type (HM715), Δ*ndh* (HM1318), Δ*oriC oriN^+^* (HM957), Δ*ndh* Δ*oriC oriN^+^* (HM1319); **(B)** P_spac_-*gapA* (HM1208), P_spac_-*gapA* Δ*oriC oriN^+^* (HM1221); **(C)** Cultures were supplemented with 0.2% sodium acetate. Wild-type (HM715), Δ*pdhB* (HM1248), Δ*oriC oriN^+^* (HM950), Δ*pdhB* Δ*oriC oriN^+^* (HM1266); **(D)** P_spac_-*fabHA* (HM964), P_spac_-*fabHA* Δ*oriC oriN^+^* (HM966); **(E)** P_xyl_-*plsC* (HM1364), P_xyl_-*plsC* Δ*oriC oriN^+^* (HM1373); **(F)** Wild-type (HM715), Δ*ltaS* (HM1168), Δ*oriC oriN^+^* (HM957), Δ*ltaS* Δ*oriC oriN^+^* (HM1244).
(PDF)

Figure S8 Analysis of *oriC*-independent growth rate regulation through genetic targeting of essential cellular activities. Strains were grown and data presented as described for Figure S7, except that the depletion of PgsA required supplementation with 1 mM IPTG to overexpress the xylose repressor. Genomic DNA was harvested from cells and marker frequency analysis was determined using qPCR. The *ori:ter* ratios are plotted versus growth rate and the percentage change in the *ori:ter* ratios comparing each deletion/depletion is indicated (error bars indicate the standard deviation of three technical replicates). Representative data are shown from a single experiment; an independently performed replicate of the experiment is shown in Figure 6. **(A)** P_spac_-*pykA* (HM1176), P_spac_-*pykA* Δ*oriC oriN^+^* (HM1186); **(B)** P_xyl_-*pgsA* (HM1365), P_xyl_-*pgsA* Δ*oriC oriN^+^* (HM1374); **(C)** Wild-type (HM715), Δ*rpsU* (HM1150), Δ*rplA* (HM1151), Δ*rplW* (HM1152), Δ*rpmJ* (HM1154), Δ*oriC oriN^+^* (HM950), Δ*rpsU* Δ*oriC oriN^+^* (HM1156), Δ*rplA* Δ*oriC oriN^+^* (HM1157), Δ*rplW* Δ*oriC oriN^+^* (HM1158), Δ*rpmJ* Δ*oriC oriN^+^* (HM1160). **(D)** P_spac_-*pykA* (HM1176), P_spac_-*pykA* Δ*dnaA oriN^+^* (HM1425); **(E)** P_xyl_-*pgsA* (HM1365), P_xyl_-*pgsA* Δ*dnaA oriN^+^* (HM1433); **(F)** Wild-type (HM715), Δ*rpsU* (HM1150), Δ*rplA* (HM1151), Δ*rpmJ* (HM1154), Δ*dnaA oriN^+^* (HM1423), Δ*rpsU* Δ*dnaA oriN^+^* (HM1429), Δ*rplA* Δ*dnaA oriN^+^* (HM1430), Δ*rpmJ* Δ*dnaA oriN^+^* (HM1432).
(PDF)

Figure S9 Analysis of *oriC*-dependent and *oriC*-independent growth rate regulation through small molecule targeting of fatty acid synthesis and protein synthesis. Strains were grown overnight at 37°C in LB medium. Overnight cultures were diluted 1∶1000 into fresh LB medium either without or with antibiotics (2 µg/ml cerulenin **(A)**, 1 µg/ml chloramphenicol **(B)**) and grown at 37°C until they reached an A_600_ of 0.3-0.5. For datapoints “+” indicates the presence of the small molecule inhibitor and “-” indicates the absence. Genomic DNA was harvested from cells and marker frequency analysis was determined using qPCR. The *ori:ter* ratios are plotted versus growth rate and the percentage change in the *ori:ter* ratios comparing each deletion/depletion is indicated (error bars indicate the standard deviation of three technical replicates). Representative data are shown from a single experiment; independently performed replicates of the experiments are shown in Figures 7A–B. Wild-type (HM715), Δ*oriC oriN^+^* (HM950), Δ*dnaA oriN^+^* (HM1423).
(PDF)

Table S1 Strain list.
(PDF)

Table S2 Plasmid list.
(PDF)

Table S3 Description of plasmids constructed and primers used.
(PDF)

Text S1 Supplementary references.
(PDF)
