# Supplementary figures and images for: Multiple Regulatory Systems Coordinate DNA Replication with Cell Growth in Bacillus subtilis
Source: PLoS Genet. 2014 Oct 23;10(10):e1004731. doi: 10.1371/journal.pgen.1004731 (PMC4207641; doi:10.1371/journal.pgen.1004731)

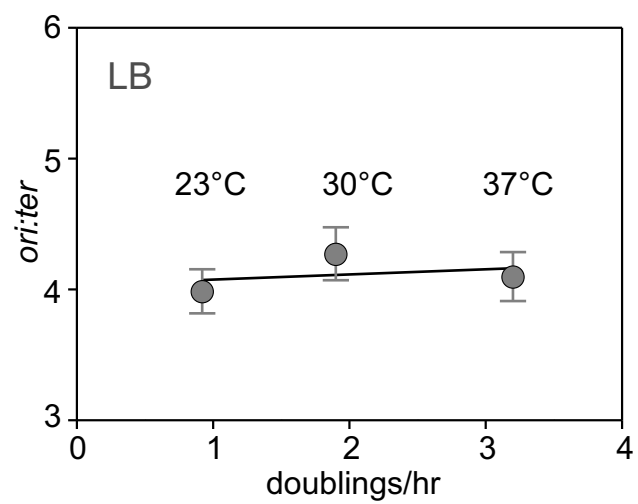

Figure S1

Supplement: Figure S1 — Culturing B. subtilis at different temperatures generates a range of steady-state growth rates but does not affect the frequency of DNA replication initiation. A wild-type strain (HM715) was grown overnight at 23°C in LB. The culture was diluted 1:100 into LB and incubated at different temperatures to generate a range of steady-state growth rates until an A600 of 0.2-0.3. Genomic DNA was harvested from cells and marker frequency analysis was determined using qPCR. The ori:ter ratios are plotted versus growth rate (error bars indicate the standard deviation of three technical replicates). Representative data are shown from a single experiment; an independently performed replicate of the experiment is shown in Figure 1B. (PDF) [file pgen.1004731.s001.pdf]

A

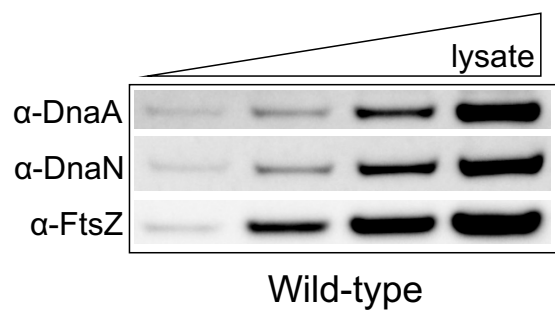

B

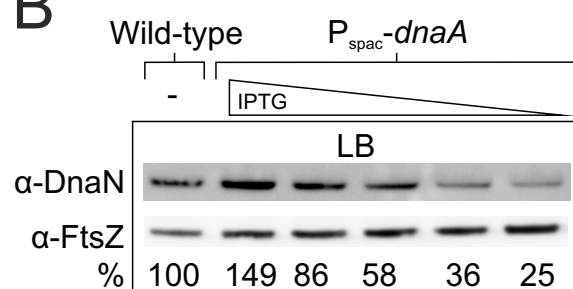

C

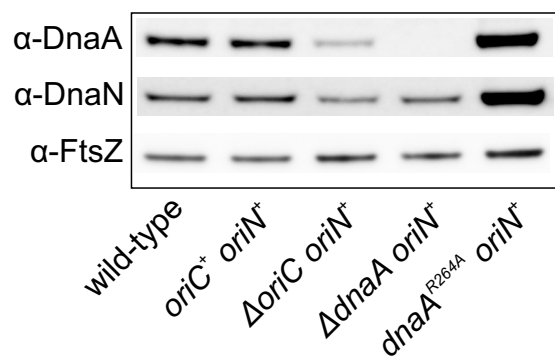

Figure S3

Supplement: Figure S3 — Western blot analysis of wild-type, oriN, and Pspac-dnaA-dnaN strains. Strains were grown overnight at 37°C in LB medium. Overnight cultures were diluted 1∶1000 into fresh LB medium and grown at 37°C until an A600 of 0.5-0.7 was attained. Cells were lysed and proteins were detected using Western blot analysis. (A) A two-fold dilution series of a cell lysate was used to determine the linear range for each antibody. (B) The endogenous dnaA-dnaN operon was placed under the control of the IPTG-inducible promoter Pspac to generate a range of expression levels. HM742 was supplemented with IPTG (400 µM) and erythromycin. The cultures were diluted 1∶100 into LB and grown at 37°C until an A600 of 0.5–0.6; HM742 was supplemented with erythromycin and a range of IPTG (800, 400, 200, 100, 50 µM). Cells were lysed and DnaN protein was detected using Western blot analysis (FtsZ protein was likewise detected and used as a loading control). The amount of DnaN was determined using densitometry; values were normalized to wild-type. Wild-type (HM222), Pspac-dnaA-dnaN (HM742). (C) Analysis of oriN strains. Wild-type (HM715), oriC+ oriN+ (HM949), ΔoriC oriN+ (HM957), ΔdnaA oriN+ (HM1423), dnaAR264A oriN+ (HM1122). (PDF) [file pgen.1004731.s003.pdf]

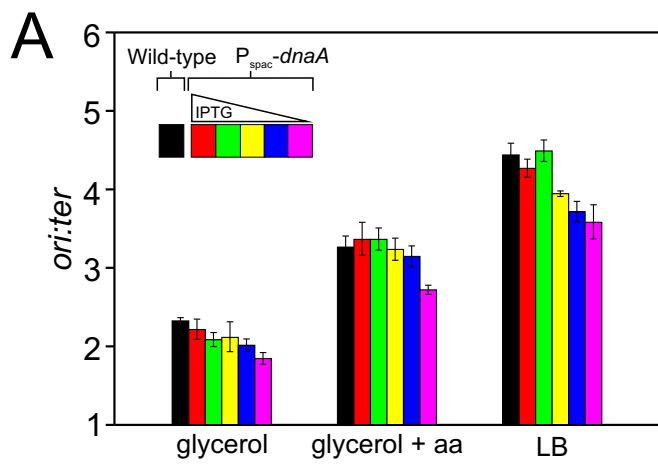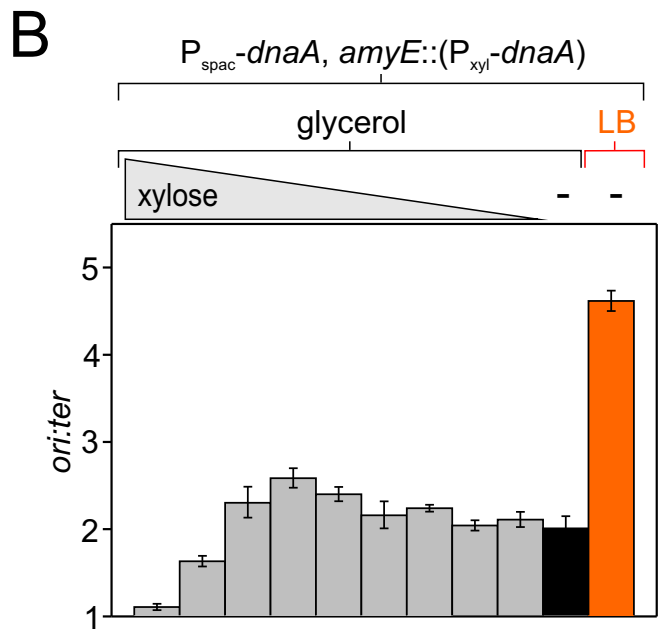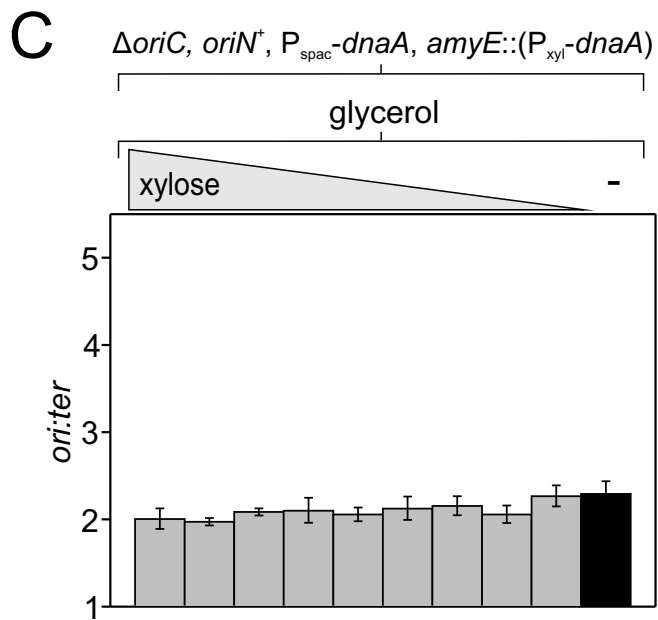

Figure S4

Supplement: Figure S4 — Changes in DnaA protein level are not sufficient to account for nutrient-mediated growth rate regulation of DNA replication initiation in B. subtilis. (A) The endogenous dnaA gene was placed under the control of the IPTG-inducible promoter Pspac to generate a range of DnaA protein levels. Strains were grown overnight at 37°C in minimal media supplemented with succinate and amino acids (20 µg/ml); IPTG (400 µM) and erythromycin was added to HM742. The cultures were diluted 1∶100 into various media (glycerol, glycerol + amino acids, LB) to generate a range of steady-state growth rates and grown at 37°C until an A600 of 0.5–0.6; in each medium HM742 was supplemented with erythromycin and a range of IPTG (800, 400, 200, 100, 50 µM). Genomic DNA was harvested from cells and marker frequency analysis was determined using qPCR. For each growth media, the ori:ter ratios are plotted versus IPTG concentration (error bars indicate the standard deviation of three technical replicates). Representative data are shown from a single experiment; an independently performed replicate of the experiment is shown in Figure 2B. Wild-type (HM222), Pspac-dnaA (HM742). (B) To strongly overexpress DnaA the endogenous dnaA gene was placed under the control of Pspac and an ectopic copy of dnaA was integrated at the amyE locus under the control of the xylose inducible promoter Pxyl (HM745). The strain was grown overnight at 37°C in minimal media supplemented with glycerol, amino acids (20 µg/ml), IPTG (800 µM), and erythromycin. The culture was diluted 1∶100 into media containing IPTG (800 µM), erythromycin, either glycerol minimal media supplemented with a range of xylose (1, 0.5, 0.25, 0.125, 0.063, 0.031, 0.016, 0.008, 0.004, 0%) or LB, and grown at 37°C until an A600 of 0.2–0.4. Genomic DNA was harvested from cells and marker frequency analysis was determined using qPCR. For each growth media, the ori:ter ratios are plotted versus xylose concentration (error bars indicate the standard deviat [file pgen.1004731.s004.pdf]

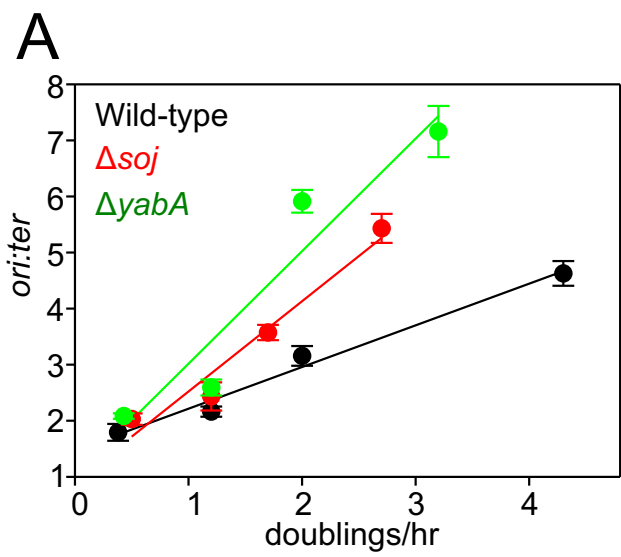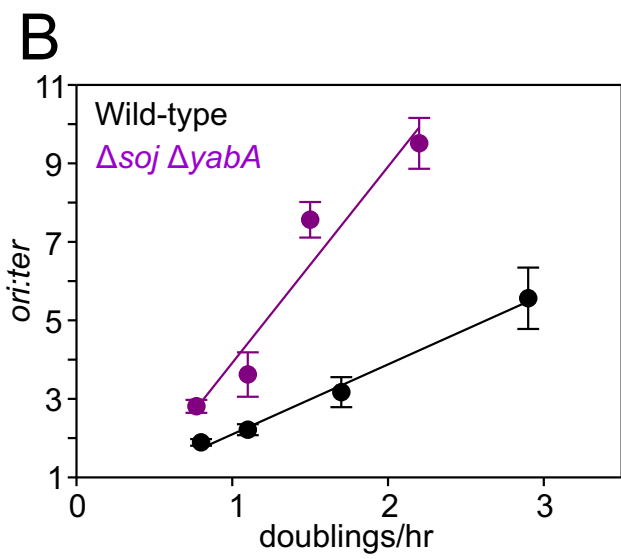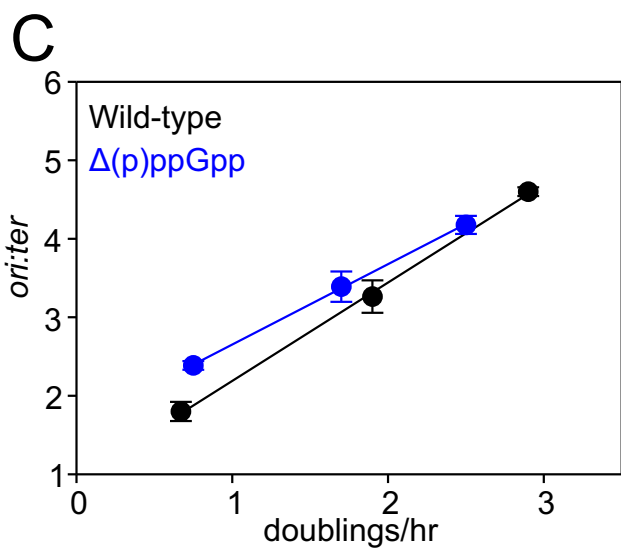

Figure S5

Supplement: Figure S5 — Nutrient-mediated growth rate regulation of DNA replication initiation is independent of Soj, YabA, and (p)ppGpp. (A) Growth rate regulation of DNA replication initiation is maintained in either Δsoj or ΔyabA mutants. Strains were grown overnight at 37°C in minimal media supplemented with succinate and amino acids (20 µg/ml). The culture was diluted 1∶100 into various media (succinate, glycerol, glycerol + amino acids, LB) to generate a range of steady-state growth rates and incubated at 37°C until an A600 of 0.3–0.4. Genomic DNA was harvested from cells and marker frequency analysis was determined using qPCR. The ori:ter ratios are plotted versus growth rate (error bars indicate the standard deviation of three technical replicates). Representative data are shown from a single experiment; an independently performed replicate of the experiment is shown in Figure 3A. Wild-type (HM222), Δsoj (HM227), ΔyabA (HM739). (B) Growth rate regulation of DNA replication initiation is maintained in a Δsoj ΔyabA double mutant. Cells were grown as in (A). Genomic DNA was harvested from cells and marker frequency analysis was determined using qPCR. The ori:ter ratios are plotted versus growth rate (error bars indicate the standard deviation of three technical replicates). Representative data are shown from a single experiment; an independently performed replicate of the experiment is shown in Figure 3A. Wild-type (HM222), Δsoj ΔyabA (HM741). (C) Growth rate regulation of DNA replication initiation does not require (p)ppGpp. Strains were grown overnight at 37°C in minimal media supplemented with succinate and amino acids (200 µg/ml). The culture was diluted 1∶100 into various media (succinate + amino acids, glycerol + amino acids, LB) to generate a range of steady-state growth rates and incubated at 37°C until an A600 of 0.2–0.6. Genomic DNA was harvested from cells and marker frequency analysis was determined using qPCR. The ori:ter ratios are plotted versus growth rate (error bars [file pgen.1004731.s005.pdf]

A

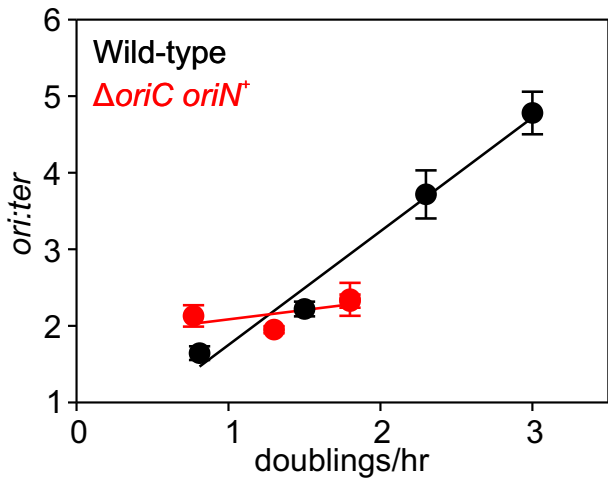

B

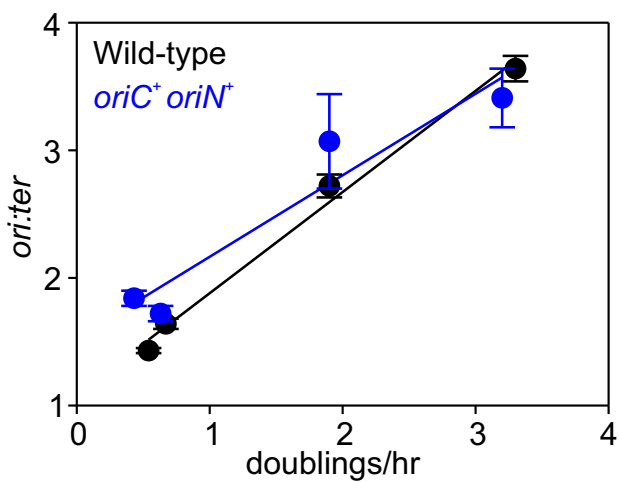

C

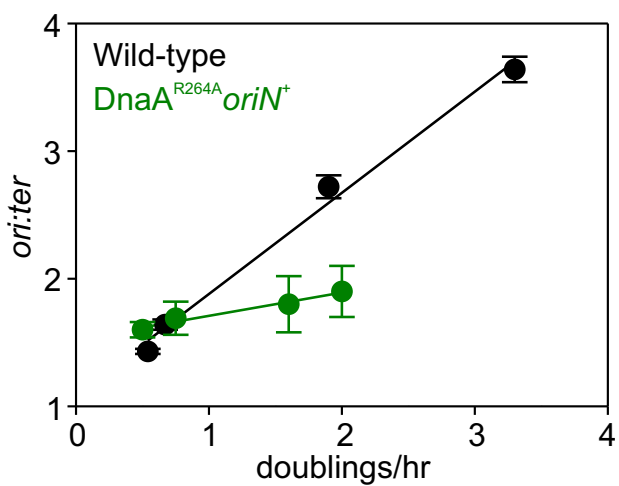

Figure S6

Supplement: Figure S6 — Nutrient-mediated growth rate regulation of DNA replication initiation requires oriC and DnaA. (A) oriC is required for growth rate regulation of DNA replication initiation. Strains were grown overnight at 37°C in minimal media supplemented with succinate and amino acids (20 µg/ml). The culture was diluted 1∶100 into various media (succinate, glycerol, glycerol + amino acids, LB) to generate a range of steady-state growth rates and incubated at 37°C until an A600 of 0.3–0.4. Genomic DNA was harvested from cells and marker frequency analysis was determined using qPCR. The ori:ter ratios are plotted versus growth rate (error bars indicate the standard deviation of three technical replicates). Representative data are shown from a single experiment; an independently performed replicate of the experiment is shown in Figure 4A. Wild-type (HM715), ΔoriC oriN+ (HM950). (B) Integration of oriN into the B. subtilis chromosome does not eliminate growth rate regulation of DNA replication initiation. Strains were grown as in (A). Genomic DNA was harvested from cells and marker frequency analysis was determined using qPCR. The ori:ter ratios are plotted versus growth rate (error bars indicate the standard deviation of three technical replicates). Representative data are shown from a single experiment; an independently performed replicate of the experiment is shown in Figure 4B. Wild-type (HM715), oriC + oriN+ (HM949). (C) DnaA activity is required for growth rate regulation of DNA replication initiation. Strains were grown as in (B). Genomic DNA was harvested from cells and marker frequency analysis was determined using qPCR. The ori:ter ratios are plotted versus growth rate (error bars indicate the standard deviation of three technical replicates). Representative data are shown from a single experiment; an independently performed replicate of the experiment is shown in Figure 4C. Wild-type (HM715), DnaAR264A oriN+ (HM1122). (PDF) [file pgen.1004731.s006.pdf]

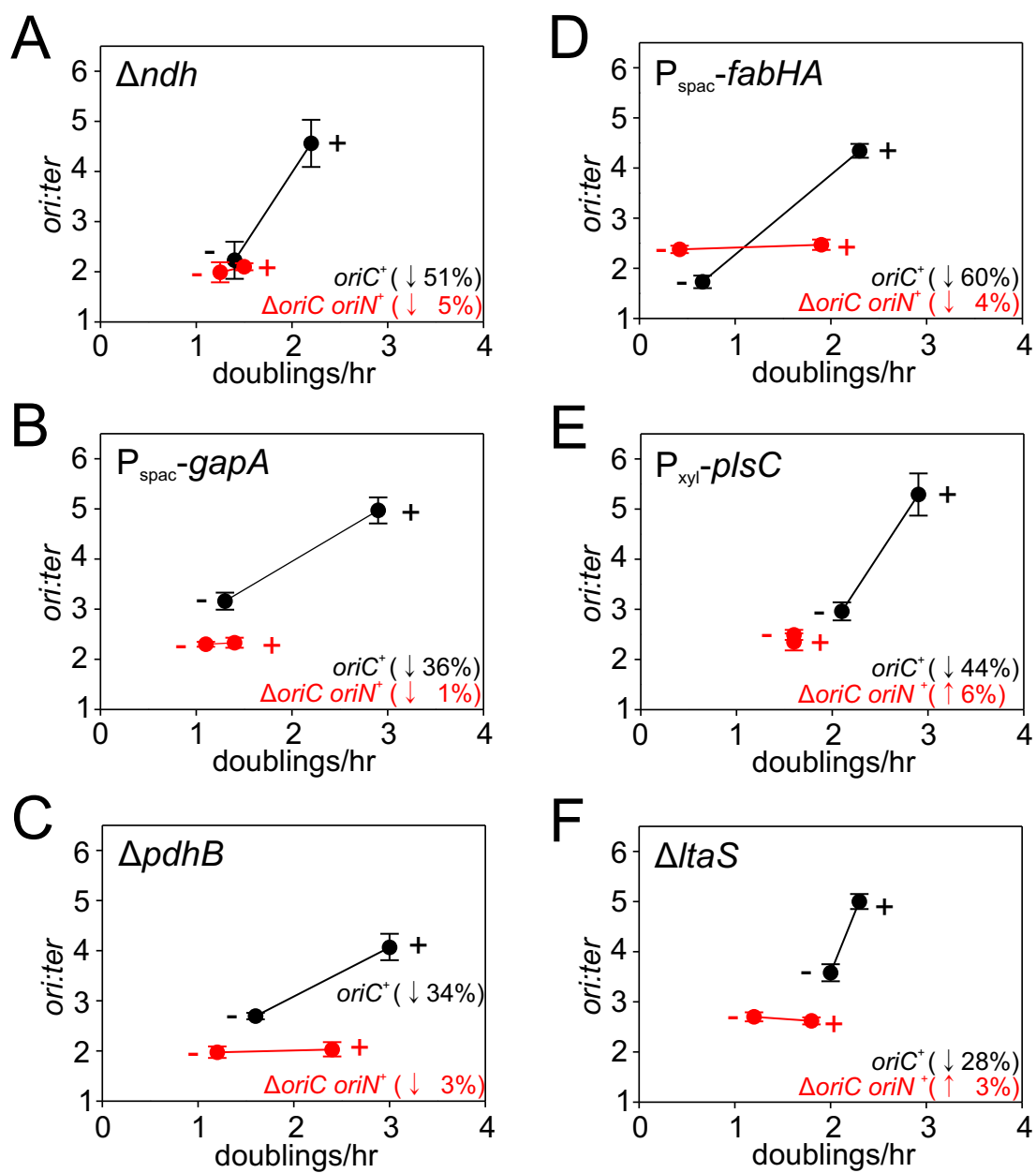

Figure S7

Supplement: Figure S7 — Analysis of oriC-dependent growth rate regulation through genetic targeting of essential cellular activities. Strains were grown overnight at 37°C in LB medium; strains harbouring plasmids integrated into the genome by single-crossover were supplemented with appropriate antibiotics and inducer (0.1 mM IPTG or 0.1% xylose). Overnight cultures were diluted 1∶1000 into fresh LB medium and grown at 37°C until they reached an A600 of 0.3–0.5; strains harbouring plasmids integrated by single-crossover were supplemented with appropriate antibiotics either without or with the appropriate inducer (1 mM IPTG or 1% xylose). For datapoints “+” indicates the presence of either the wild-type gene (when comparing with knockout mutants) or the inducer; “−” indicates the absence of either the gene (when comparing with wild-type) or the inducer. Genomic DNA was harvested from cells and marker frequency analysis was determined using qPCR. The ori:ter ratios are plotted versus growth rate and the percentage change in the ori:ter ratios comparing each deletion/depletion is indicated (error bars indicate the standard deviation of three technical replicates). Representative data are shown from a single experiment; an independently performed replicate of the experiment is shown in Figure 5. (A) Wild-type (HM715), Δndh (HM1318), ΔoriC oriN+ (HM957), Δndh ΔoriC oriN+ (HM1319); (B) Pspac-gapA (HM1208), Pspac-gapA ΔoriC oriN+ (HM1221); (C) Cultures were supplemented with 0.2% sodium acetate. Wild-type (HM715), ΔpdhB (HM1248), ΔoriC oriN+ (HM950), ΔpdhB ΔoriC oriN+ (HM1266); (D) Pspac-fabHA (HM964), Pspac-fabHA ΔoriC oriN+ (HM966); (E) Pxyl-plsC (HM1364), Pxyl-plsC ΔoriC oriN+ (HM1373); (F) Wild-type (HM715), ΔltaS (HM1168), ΔoriC oriN+ (HM957), ΔltaS ΔoriC oriN+ (HM1244). (PDF) [file pgen.1004731.s007.pdf]

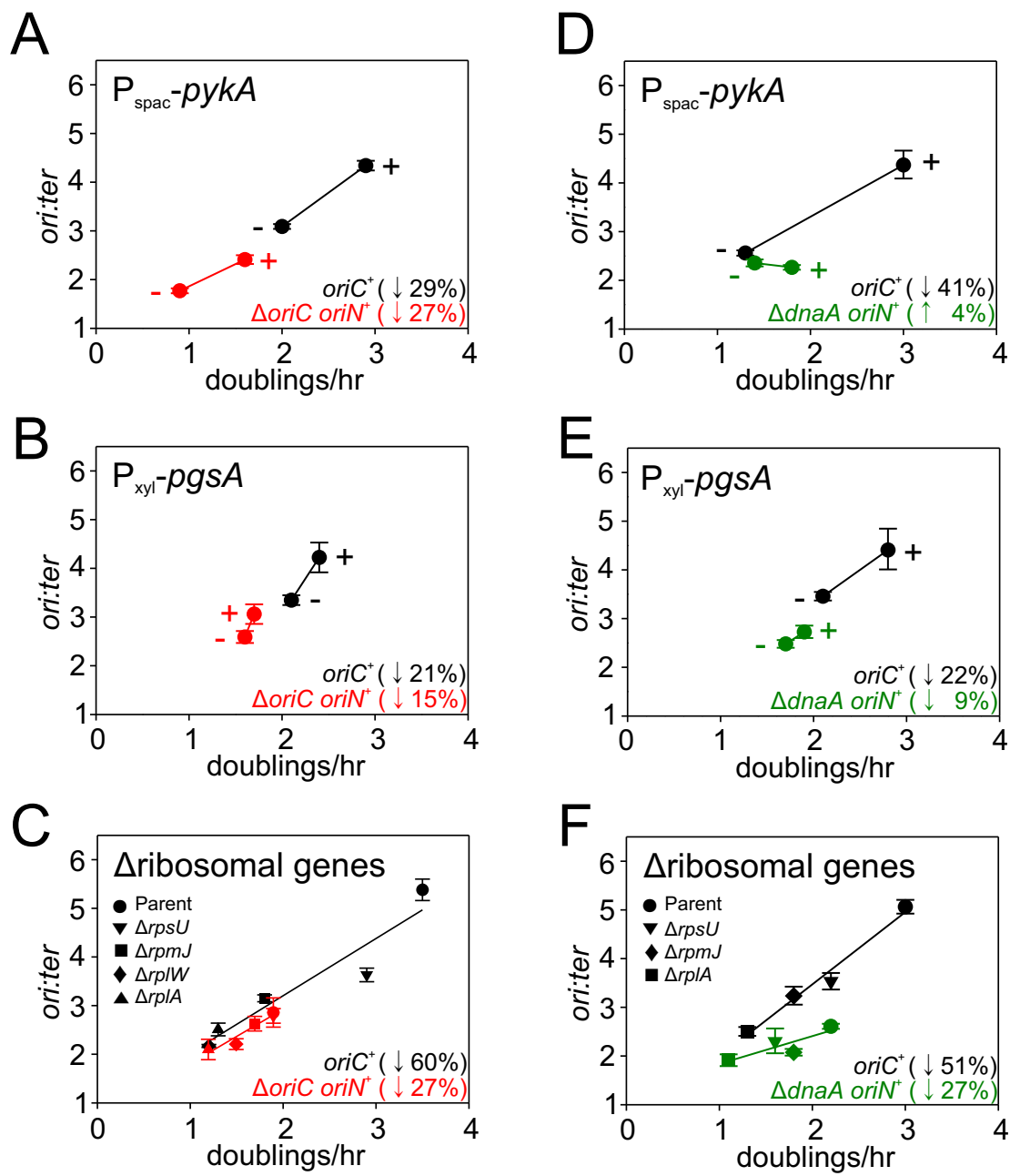

Figure S8

Supplement: Figure S8 — Analysis of oriC-independent growth rate regulation through genetic targeting of essential cellular activities. Strains were grown and data presented as described for Figure S7, except that the depletion of PgsA required supplementation with 1 mM IPTG to overexpress the xylose repressor. Genomic DNA was harvested from cells and marker frequency analysis was determined using qPCR. The ori:ter ratios are plotted versus growth rate and the percentage change in the ori:ter ratios comparing each deletion/depletion is indicated (error bars indicate the standard deviation of three technical replicates). Representative data are shown from a single experiment; an independently performed replicate of the experiment is shown in Figure 6. (A) Pspac-pykA (HM1176), Pspac-pykA ΔoriC oriN+ (HM1186); (B) Pxyl-pgsA (HM1365), Pxyl-pgsA ΔoriC oriN+ (HM1374); (C) Wild-type (HM715), ΔrpsU (HM1150), ΔrplA (HM1151), ΔrplW (HM1152), ΔrpmJ (HM1154), ΔoriC oriN+ (HM950), ΔrpsU ΔoriC oriN+ (HM1156), ΔrplA ΔoriC oriN+ (HM1157), ΔrplW ΔoriC oriN+ (HM1158), ΔrpmJ ΔoriC oriN+ (HM1160). (D) Pspac-pykA (HM1176), Pspac-pykA ΔdnaA oriN+ (HM1425); (E) Pxyl-pgsA (HM1365), Pxyl-pgsA ΔdnaA oriN+ (HM1433); (F) Wild-type (HM715), ΔrpsU (HM1150), ΔrplA (HM1151), ΔrpmJ (HM1154), ΔdnaA oriN+ (HM1423), ΔrpsU ΔdnaA oriN+ (HM1429), ΔrplA ΔdnaA oriN+ (HM1430), ΔrpmJ ΔdnaA oriN+ (HM1432). (PDF) [file pgen.1004731.s008.pdf]

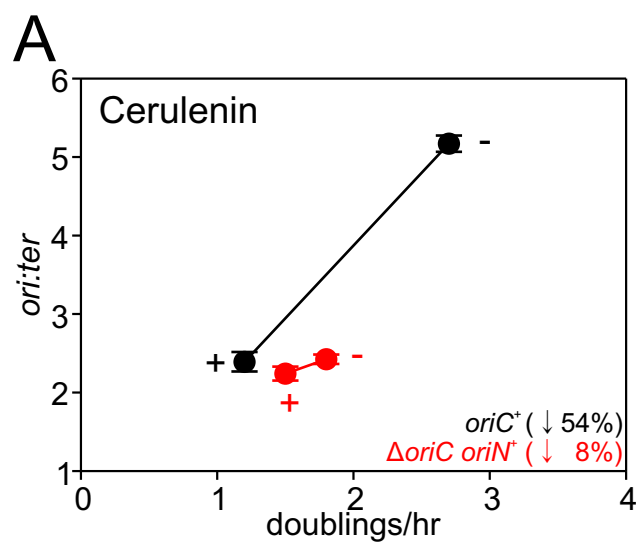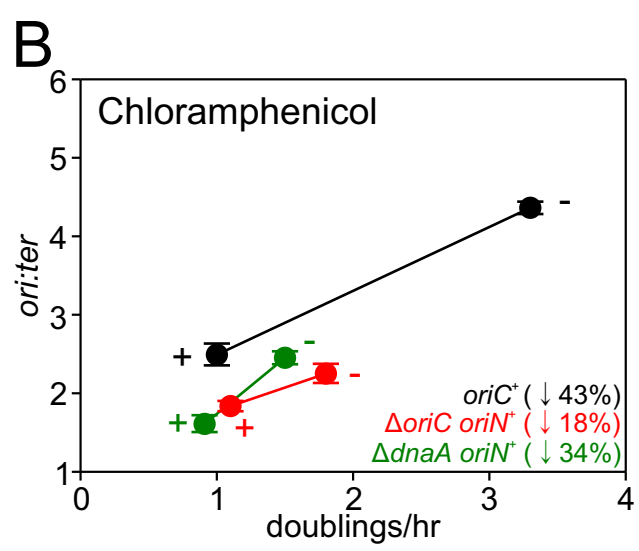

Figure S9

Supplement: Figure S9 — Analysis of oriC-dependent and oriC-independent growth rate regulation through small molecule targeting of fatty acid synthesis and protein synthesis. Strains were grown overnight at 37°C in LB medium. Overnight cultures were diluted 1∶1000 into fresh LB medium either without or with antibiotics (2 µg/ml cerulenin (A), 1 µg/ml chloramphenicol (B)) and grown at 37°C until they reached an A600 of 0.3-0.5. For datapoints “+” indicates the presence of the small molecule inhibitor and “-” indicates the absence. Genomic DNA was harvested from cells and marker frequency analysis was determined using qPCR. The ori:ter ratios are plotted versus growth rate and the percentage change in the ori:ter ratios comparing each deletion/depletion is indicated (error bars indicate the standard deviation of three technical replicates). Representative data are shown from a single experiment; independently performed replicates of the experiments are shown in Figures 7A–B. Wild-type (HM715), ΔoriC oriN+ (HM950), ΔdnaA oriN+ (HM1423). (PDF) [file pgen.1004731.s009.pdf]
