# Supplementary material for: Multiple Regulatory Systems Coordinate DNA Replication with Cell Growth in Bacillus subtilis
Source: PLoS Genet. 2014 Oct 23;10(10):e1004731. doi: 10.1371/journal.pgen.1004731 (PMC4207641; doi:10.1371/journal.pgen.1004731)
Supplement: Figure S2 — Cell measurements as a function of nutrient-mediated growth rate. (A) Measurement of replication origins per cell. An array of ∼25 tetO sites was inserted near the replication origin and was visualized using TetR-GFP. Strain AK47 was grown overnight at 37°C in minimal media supplemented with succinate (2%), amino acids (0.2%), spectinomycin (50 µg/ml) and erythromycin (1 µg/ml). Cultures were washed twice and diluted 1∶100 into various chemically defined media supplemented with either succinate (2%), glucose (1.5%), or glucose (1.5%) with amino acids (200 µg/ml) and grown at 37°C until they reached an A600 of 0.3–0.5. Samples were taken for microscopy and membranes were stained. Scale bar represents 3 µm. (B) Quantification of the number of origins per cell at different growth rates. The average number of origins per cell is indicated above each histogram. (C,E) Cell lengths were grouped according to the number of origins, measurements were binned in 0.5 µm steps, and data plotted as a percentage within each population. (D,F) The average cell lengths and widths (+/− standard deviation) were grouped according to the number of origins per cell. (PDF) [file pgen.1004731.s002.pdf]

A

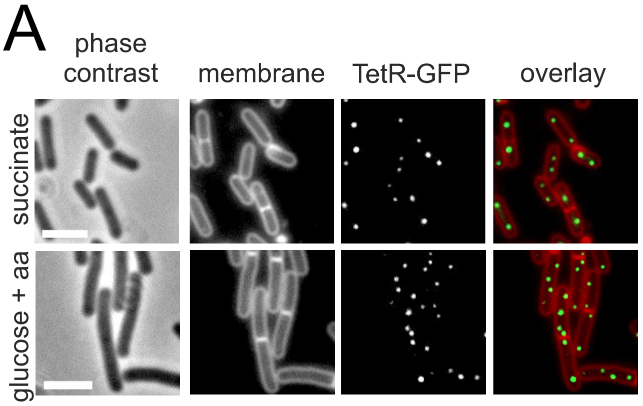

B

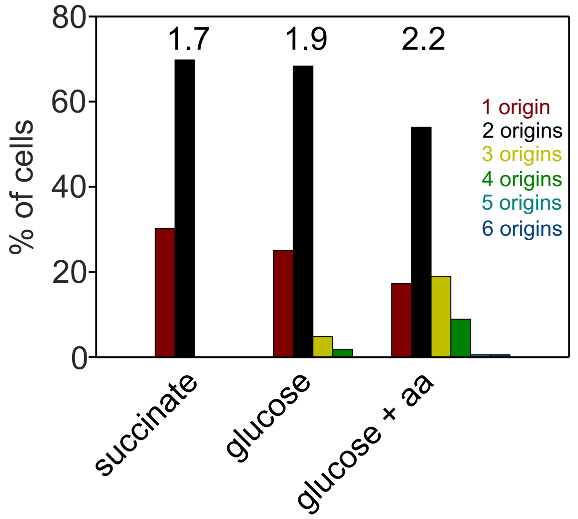

C

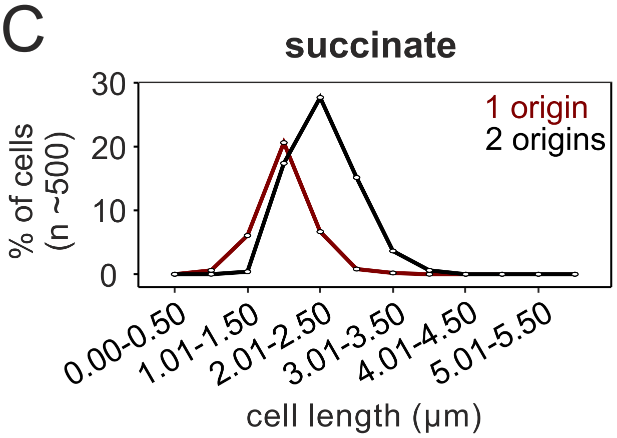

D

Cell length & width in succinate media (n~500)		
	Average (µm) ± SD	
no. of origin (s)	Length	Width
1	1.77 ± 0.35	0.77 ± 0.09
2	2.30 ± 0.44	0.78 ± 0.09

E

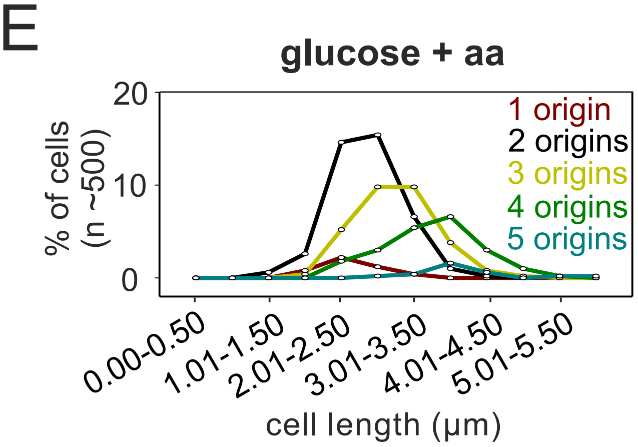

F

Cell length & width in glucose + aa media (n~500)		
	Average (µm) ± SD	
no. of origin (s)	Length	Width
1	2.34 ± 0.42	0.74 ± 0.09
2	2.64 ± 0.49	0.74 ± 0.10
3	3.09 ± 0.51	0.76 ± 0.10
4	3.52 ± 0.64	0.77 ± 0.10
5	4.77 ± 0.87	0.84 ± 0.02

Figure S2
