# Supplementary material for: Multiple Regulatory Systems Coordinate DNA Replication with Cell Growth in Bacillus subtilis
Source: PLoS Genet. 2014 Oct 23;10(10):e1004731. doi: 10.1371/journal.pgen.1004731 (PMC4207641; doi:10.1371/journal.pgen.1004731)
Supplement: Table S1 — Strain list. (PDF) [file pgen.1004731.s010.pdf]

Table S1: Strain list

Strain	Genotype	Reference
168CA	*trpC2*	[46]
4284	*trpC2* Δ*ltaS*::*spc*	[47]
1A1066	*trpC2* Δ*pdhB*::*spc*	Bacillus Genetic Stock Center
AK47	*trpC2 yycR*::($tetO_{25}$ *erm*) *amyE*::($P_{spac(c)}$-*tetR-gfp spc*)	this work
AK647	*trpC2 pheA1 spo0J*::($tetO_{150}$ *neo*) *cgeD*::($P_{pen(mutTTAG)}$-*tetR-yfp tet*)	this work
AK652	*trpC2 pheA1 dnaA'*Ω(P_{spac}-*dnaA-dnaN erm*) *spo0J*::($tetO_{150}$ *neo*) *cgeD*::($P_{pen(mutTTAG)}$-*tetR-yfp tet*)	this work
BKM865	*yycR*::($tetO_{25}$ *cat*) *amyE*::($P_{spac(c)}$-*tetR-gfp spc*)	[48]
BKM918	*yycR*::($tetO_{25}$ *erm*)	[48]
bSS186	Δ*relA*::*mls* Δ*yjbM*::*cat* Δ*ywaC*::*spc* *amyE*::*tetL*(P_{rpsD}-*gfp-ssrA)*	gift from S. Syvertsson
HM130	*trpC2 spo0J*::($tetO_{150}$ *neo*) *cgeD*::($P_{pen(mutTTAG)}$-*tetR-yfp tet*)	[21]
HM222	*trpC2 pheA1 spo0J*::*neo*	[21]
HM227	*trpC2 pheA1* Δ*soj*::*neo*	[21]
HM228	*pheA1* (*ypjG-hepT*)22 *spoIIIJ::pRK1(oriN cat)* Δ*oriC-S*	[21]
HM259	*trpC2 dnaA'*Ω(P_{spac}-*dnaA erm*) Δ*soj*::*neo* *amyE*::(Pxyl-soj^{G12V} *spc*)	[21]
HM715	*trpC2* (laboratory strain 168CA)	[46]
HM739	*trpC2 pheA1 spo0J*::*neo* Δ*yabA*::*phleo*	this work
HM741	*trpC2 pheA1* Δ*soj*::*neo* Δ*yabA*::*phleo*	this work
HM742	*trpC2 pheA1 dnaA'*Ω(P_{spac}-*dnaA erm*)	this work

HM745	*trpC2 pheA1 dnaA'*Ω(P_{spac}-*dnaA erm*) *amyE*::(P_{xyl}-*dnaA cat*)	this work
HM949	*trpC2 spoIIIJ*::(*oriN kan tet*)	this work
HM950	*trpC2 spoIIIJ*::(*oriN kan tet*) Δ*oriC-S*	this work
HM957	*trpC2 spoIIIJ*::(*oriN cat*) Δ*oriC-S*	this work
HM963	*trpC2 spoIIIJ*::(*oriN kan tet*) Δ*incAB*::(P_{spac}-*dnaA erm*)	this work
HM964	*trpC2 fabHA'*Ω(P_{spac}-*fabHA erm*)	this work
HM966	*trpC2 spoIIIJ*::(*oriN kan tet*) Δ*oriC-S fabHA'*Ω(P_{spac}-*fabHA erm*)	this work
HM1080	*trpC2 plsC'*Ω(P_{xyl}-*plsC spc*) *amyE*::(P_{xyl}-*xylR tet*)	this work
HM1086	*trpC2 spoIIIJ*::(*oriN cat*) Δ*oriC-S plsC'*Ω(P_{xyl}-*plsC spc*) *amyE*::(P_{xyl}-*xylR tet*)	this work
HM1122	*trpC2 spoIIIJ*::(*oriN kan tet*) $dnaA^{R264A}$::*cat*	this work
HM1150	*trpC2* Δ*rpsU*::*cat*	this work
HM1151	*trpC2* Δ*rplA*::*cat*	this work
HM1152	*trpC2* Δ*rplW*::*cat*	this work
HM1154	*trpC2* Δ*rpmJ*::*cat*	this work
HM1156	*trpC2 spoIIIJ*::(*oriN kan tet*) Δ*oriC-S* Δ*rpsU*::*cat*	this work
HM1157	*trpC2 spoIIIJ*::(*oriN kan tet*) Δ*oriC-S* Δ*rplA*::*cat*	this work
HM1158	*trpC2 spoIIIJ*::(*oriN kan tet*) Δ*oriC-S* Δ*rplW*::*cat*	this work
HM1160	*trpC2 spoIIIJ*::(*oriN kan tet*) Δ*oriC-S* Δ*rpmJ*::*cat*	this work
HM1168	*trpC2* Δ*ltaS::spc*	this work
HM1176	*trpC2 pykA'*Ω(P_{spac}-*pykA erm*)	this work
HM1186	*trpC2 spoIIIJ*::(*oriN kan tet*) Δ*oriC-S pykA'*Ω(P_{spac}-*pykA erm*)	this work
HM1208	*trpC2 gapA'*Ω(P_{spac}-*gapA erm*) *amyE*::($P_{spac\text{-}hy}$-*gapA cat*)	this work

HM1221	*trpC2 spoIIIJ*::(*oriN kan tet*) Δ*oriC-S gapA'*Ω(P_{spac}-*gapA erm*) *amyE::*($P_{spac-hy}$-*gapA cat*)	this work
HM1230	*trpC2 pheA1* Δ*relA*::*mls* Δ*yjbM*::*cat* Δ*ywaC*::*spc spo0J*::*neo*	this work
HM1244	*trpC2 spoIIIJ*::(*oriN cat*) Δ*oriC-S* Δ*ltaS*::*spc*	this work
HM1248	*trpC2* Δ*pdhB*::*spc*	this work
HM1266	*trpC2 spoIIIJ*::(*oriN kan tet*) Δ*oriC-S* Δ*pdhB*::*spc*	this work
HM1318	*trpC2* Δ*ndh*::*kan*	this work
HM1319	*trpC2 spoIIIJ*::(*oriN cat*) Δ*oriC-S* Δ*ndh*::*kan*	this work
HM1364	*trpC2 plsC'*Ω(P_{xyl}-*plsC spc*) *aprE*::(P_{spac}-P_{xyl}-*xylR lacI erm*)	this work
HM1365	*trpC2 pgsA'*Ω(P_{xyl}-*pgsA cat*) *aprE*::(P_{spac}-P_{xyl}-*xylR lacI erm*)	this work
HM1373	*trpC2 spoIIIJ*::(*oriN kan tet*) Δ*oriC-S plsC'*Ω(P_{xyl}-*plsC spc*) *aprE*::(P_{spac}-P_{xyl}-*xylR lacI erm*)	this work
HM1374	*trpC2 spoIIIJ*::(*oriN kan tet*) Δ*oriC-S pgsA'*Ω(P_{xyl}-*pgsA cat*) *aprE*::(P_{spac}-P_{xyl}-*xylR lacI erm*)	this work
HM1423	*trpC2 spoIIIJ*::(*oriN kan tet*) Δ*dnaA*::*zeo*	this work
HM1425	*trpC2 spoIIIJ*::(*oriN kan tet*) Δ*dnaA*::*zeo pykA'*Ω(P_{spac}-*pykA erm*)	this work
HM1429	*trpC2 spoIIIJ*::(*oriN kan tet*) Δ*dnaA*::zeo Δ*rpsU*::*cat*	this work
HM1430	*trpC2 spoIIIJ*::(*oriN kan tet*) Δ*dnaA*::*zeo* Δ*rplA*::*cat*	this work
HM1432	*trpC2 spoIIIJ*::(*oriN kan tet*) Δ*dnaA*::*zeo* Δ*rpmJ*::*cat*	this work
HM1433	*trpC2 spoIIIJ*::(*oriN kan tet*) Δ*dnaA*::*zeo pgsA'*Ω(P_{xyl}-*pgsA cat*) *aprE*::(P_{spac}-P_{xyl}-*xylR lacI erm*)	this work
HM1467	*trpC2 spoIIIJ*::(*oriN kan tet*) Δ*incAB*::(P_{spac}-*dnaA erm*) *amyE*::(P_{xyl}-*dnaA cat*)	this work
JH642	*trpC2 pheA1*	[49]

MBS17	*trpC2* Δ*rpsU*::*cat*	[50]
MBS18	*trpC2* Δ*rplA*::*cat*	[50]
MBS22	*trpC2* Δ*rplW*::*cat*	[50]
MBS30	*trpC2* Δ*rpmJ*::*cat*	[50]
MMB208	*pheA1* (*ypjG-hepT*)22 *spoIIIJ*::(*oriN kan tet*) Δ*oriC-S*	[51]
NIS6301	*purA16 metB5 hisA3 guaB spoIIIJ*::pRK1(*oriN cat*) Δ*oriC-1*	[52]
NR19	*trpC2* Δ*yabA*::*phleo*	[26]
PY79	prototroph	[53]
RIK908	*trpC2* Δ*ywaC*::*spc*	[54]
RIK909	*trpC2* Δ*yjbm*::*spc*	[54]
RM250	*trpC2 spoVD'*Ω(P_{xyl}-*murE cat*) *amyE*::(P_{xyl}-*xylR tet) xseB** (Frameshift 22T>-) *fabHA'Ω*(P_{spac}-*fabHA erm)*	[55]
TW20	*trpC2 pheA1* Δ*relA*::*spc amyE*::(P_{spac}-*relA cat*)	[56]
UC6002A	*purA16 metB5 hisA3 guaB33 amyE*::(P_{xyl}-*dnaA cat*)	[57]
YK1714	*trpC2* Δ*ndh*::(TnYLB-1 *kan*)	gift from Y. Kawai
