# Supplementary material for: Multiple Regulatory Systems Coordinate DNA Replication with Cell Growth in Bacillus subtilis
Source: PLoS Genet. 2014 Oct 23;10(10):e1004731. doi: 10.1371/journal.pgen.1004731 (PMC4207641; doi:10.1371/journal.pgen.1004731)
Supplement: Table S2 — Plasmid list. (PDF) [file pgen.1004731.s011.pdf]

Table S2: Plasmid list

Plasmid	Genotype	Reference
pHM327	*bla dnaA*$^{ATG\text{->}TAA}$ *dnaN cat recF*	[22]
pHM453	*bla rpnA' rpmH erm* Δ*incAB* P$_{spac}$-*dnaA'*	this work
pHM455	*bla spc* P$_{xyl}$ *amyE'*	this work
pHM480	*bla spc* P$_{xyl}$-*plsC'*	this work
pHM481	*bla cat* P$_{xyl}$-*pgsA'*	this work
pHM493	*bla dnaA*$^{ATG\text{->}TAA,R264A}$ *dnaN cat recF*	this work
pHM509	*bla aprE*::(P$_{spac}$-P$_{xyl}$-*xylR lacI erm*)	this work
pHM527	*bla* P$_{dnaA}$-Δ*dnaA dnaN zeo recF*	this work
pMUTIN4	*bla erm* P$_{spac}$ *lacZ lacI*	[58]
pPL82	*bla amyE*::(*lacZ lacI* P$_{spac\text{-}hy}$ *cat*)	[59]
pRD96	*bla cat* P$_{xyl}$	[60]
pPL82-*gapA*	*bla amyE*::(*lacZ lacI* P$_{spac\text{-}hy}$-*gapA cat*)	gift from Y. Kawai
pMUTIN4-*gapA'*	*bla erm* P$_{spac}$-*gapA' lacZ lacI*	gift from Y. Kawai
pMUTIN4-*pykA'*	*bla erm* P$_{spac}$-*pykA' lacZ lacI*	gift from R. Mercier
