# Supplementary material for: Multiple Regulatory Systems Coordinate DNA Replication with Cell Growth in Bacillus subtilis
Source: PLoS Genet. 2014 Oct 23;10(10):e1004731. doi: 10.1371/journal.pgen.1004731 (PMC4207641; doi:10.1371/journal.pgen.1004731)
Supplement: Table S3 — Description of plasmids constructed and primers used. (PDF) [file pgen.1004731.s012.pdf]

PGENETICS-D-14-00610R3

Multiple regulatory systems coordinate DNA replication with cell growth in *Bacillus subtilis*

Heath Murray and Alan Koh

Plasmid construction

pHM453 [*bla rpnA' rpmH erm* Δ*incAB* P_{spac}-*dnaA'*] was created in multiple steps. First, pJS1 was generated by ligation with a HindIII-BamHI PCR product containing 5' end of *dnaA* (dnaA-F + dnaA-R and 168CA genomic DNA as template) and pMUTIN4 cut with HindIII-BamHI. Second, pHM396 was generated by digestion of pJS1 with PvuII (to remove *lacZ and lacI*) and ligation of the vector backbone. Finally, pHM453 was generated by ligation with an AatII PCR product containing *rpmH* and the 5' end of *rpnA* (oHM319 + oHM320 and 168CA genomic DNA as template) and pHM396 cut with AatII.

pHM455 [*bla spc* P_{xyl} *amyE'*] was generated by digestion of pSG1728 [1] with SmaI-MscI (to remove *'amyE*) and ligation of the vector backbone.

pHM480 [*bla spc* P_{xyl}-*plsC'*] was generated by ligation with an Asp718I-AatII PCR product containing 5' end of *plsC* (oHM426 + oHM428 and 168CA genomic DNA as template) and pHM455 cut with Asp718I-AatII.

pHM481 [*bla spc* P_{xyl}-*pgsA'*] was generated by ligation with an XbaI-HindIII PCR product containing 5' end of *pgsA* (oHM417 + oHM423 and 168CA genomic DNA as template) and pRD96 [2] cut with XbaI-HindIII.

pHM493 [*bla* $dnaA^{ATG\text{->}TAA,R264A}$ *dnaN cat recF*] was generated by Quickchange mutagenesis using oGS81 + oGS82 and pHM327 [3] as template.

pHM509 [*bla aprE*::(P_{spac}-P_{xyl}-*xylR lacI erm)*] was created in multiple steps. First, pHM294 was generated by ligation with a BlnI-NotI PCR product containing P_{xylR}-*xylR* (oHM246 + oHM247 and 168CA genomic DNA as template) and pLOSS [4] cut with BlnI-NotI. Second, pHM509 was generated by ligation of an Asp718I-XbaI DNA fragment containing P_{xylR}-*xylR* (digestion of pHM294) and pAPNC213 [5] cut with Asp718I-XbaI.

pHM527 [*bla* P_{dnaA}-Δ*dnaA dnaN zeo recF*] was created in multiple steps. First, pHM361 was generated by ligation with an Asp718I-NruI PCR product containing 5' end of *dnaA* (oHM300 + oHM301 and 168CA genomic DNA as template) and pSG1154 [1]. Second, pHM362 was generated by ligation with an AatII-SpeI PCR product containing *dnaN-cat-recF* (oHM302 + oHM303 and HM557 genomic DNA as template [3]) and pHM361 cut with AatII-SpeI. Third, pHM525 was generated by ligation of a SacI DNA fragment containing *zeo* (digestion of p7Z6 [6]) and pHM362 cut with SacI. Finally, pHM527 was generated by ligation with a NruI-SpeI PCR product containing *incAB* (oHM475 + oHM476 and 168CA genomic DNA as template) and pHM525 cut with NruI-SpeI.

pPL82-*gapA* [*bla amyE*::(*lacZ lacI* $P_{spac\text{-}hy}$-*gapA cat*)] was generated by ligation with an SphI-XbaI PCR product containing *gapA* (gapA-F1 + gapA-R1 and 168CA genomic DNA as template) and pPL82 [7].

pMUTIN4-*gapA'* [*bla erm* P_{spac}-*gapA' lacZ lacI*] was generated by ligation with an EcoRI-BamHI PCR product containing the 5' end of *gapA* (gapA-F2 + gapA-R2 and 168CA genomic DNA as template) and pMUTIN4 cut with EcoRI-BamHI.

pMUTIN4-*pykA'* [*bla erm* P_{spac}-*pykA' lacZ lacI*] was generated by ligation with an BamHI-HindIII PCR product containing the 5' end of *pykA* (pykA-F + pykA-R and 168CA genomic DNA as template) and pMUTIN4 cut with BamHI-HindIII.

Table S3: Plasmid list

Name	Sequence (5’ → 3’)
dnaA-F	CGCAAGCTTCTAAGAAAAGGAGGGACG
dnaA-R	CGGTTTCCGGATCCGATGAC
gapA-F1	TCTTCTAGATCTCTCACTTATTTAAAGG
gapA-R1	GCAGCATGCTCGAAAGAACCAAGTCAGG
gapA-F2	GGGGAATTCTCTCTCACTTATTTAAAGGAG
gapA-R2	GGGGGATCCAACGCCTTGTTTGCCCCAG
oGS81	CTTGAAGACAGATTGCGCTCAGCTTTTGAATGGGGACTTATTAC
oGS82	GTAATAAGTCCCCATTCAAAAGCTGAGCGCAATCTGTCTTCAAG
oHM246	TTCGCAAGAAGCGGCCGCATGAGATTGAGCCATGTGATTTCC
oHM247	GGATCCCTAGGAATTACATTGTAATCATGTCCAG
oHM300	CCAATTCCCCATGGTCTGATTTTCCTCAAAATATGCTC
oHM301	ACGTGGTACCCGCTTTAAGCTGTTCTTTAATTTC
oHM302	GGGAAAACTAGTCAGGACCGGGGATCAATCGGGGA
oHM303	TTTAAGGGACGTCAGCCCTCTTGAGATGCCTGCATGG
oHM319	AATAATGACGTCGGCAAATTGTTTGAATTTGTC
oHM320	AATAATAGACGTCAGCCCGACACGCAGTTCATC
oHM417	AATAATTCTAGATTACCAGATGACTTTTCTTCACGTCC
oHM423	AATAATAAGCTTCGGATACGAGCAGTTTGTCCGC
oHM426	AATAATGGTACCGATTACGACAAAGGAAGTGCG
oHM428	AATAATGACGTCCTTTCAGCAGCTTAATCGGCG
oHM475	AATAATTCGCGATTTCTTGCAACCATAATAGG
oHM476	AATAATACTAGTCTTCCGGCACGTCCC
pkyA-F	GGGAAGCTTAAGGCTGAAGATTTCAGAAGG
pkyA-R	GGGGATCCGCTCTTTGCCTGTTTCAAGC

References

1. Lewis PJ, Marston AL (1999) GFP vectors for controlled expression and dual labelling of protein fusions in *Bacillus subtilis*. Gene 227: 101-109.
2. Daniel RA, Harry EJ, Katis VL, Wake RG, Errington J (1998) Characterization of the essential cell division gene *ftsL* (*yllD*) of *Bacillus subtilis* and its role in the assembly of the division apparatus. Mol Microbiol 29: 593-604.
3. Scholefield G, Errington J, Murray H (2012) Soj/ParA stalls DNA replication by inhibiting helix formation of the initiator protein DnaA. EMBO J 31: 1542-1555.
4. Claessen D, Emmins R, Hamoen LW, Daniel RA, Errington J, et al. (2008) Control of the cell elongation-division cycle by shuttling of PBP1 protein in *Bacillus subtilis*. Mol Microbiol 68: 1029-1046.
5. Morimoto T, Loh PC, Hirai T, Asai K, Kobayashi K, et al. (2002) Six GTP-binding proteins of the Era/Obg family are essential for cell growth in *Bacillus subtilis*. Microbiology (Reading, England) 148: 3539-3552.
6. Yan X, Yu HJ, Hong Q, Li SP (2008) Cre/*lox* system and PCR-based genome engineering in *Bacillus subtilis*. Applied Environ Microbiol 74: 5556-5562.
7. Quisel JD, Burkholder WF, Grossman AD (2001) In vivo effects of sporulation kinases on mutant Spo0A proteins in *Bacillus subtilis*. J Bacteriol 183: 6573-6578.
