# Supplementary material for: Multiple Regulatory Systems Coordinate DNA Replication with Cell Growth in Bacillus subtilis
Source: PLoS Genet. 2014 Oct 23;10(10):e1004731. doi: 10.1371/journal.pgen.1004731 (PMC4207641; doi:10.1371/journal.pgen.1004731)
Supplement: Text S1 — Supplementary references. (PDF) [file pgen.1004731.s013.pdf]

Supplemental References

46. Kunst F, al. e (1997) The complete genome sequence of the Gram positive bacterium *Bacillus subtilis*. Nature 390: 249-256.
47. Schirner K, Marles-Wright J, Lewis RJ, Errington J (2009) Distinct and essential morphogenic functions for wall- and lipo-teichoic acids in *Bacillus subtilis*. EMBO J 28: 830-842.
48. Wagner JK, Marquis KA, Rudner DZ (2009) SirA enforces diploidy by inhibiting the replication initiator DnaA during spore formation in *Bacillus subtilis*. Mol Microbiol 73: 963-974.
49. Perego M, Spiegelman GB, Hoch JA (1988) Structure of the gene for the transition state regulator, *abrB*: regulator synthesis is controlled by the *spo0A* sporulation gene in *Bacillus subtilis*. Mol Microbiol 2: 689-699.
50. Akanuma G, Nanamiya H, Natori Y, Yano K, Suzuki S, et al. (2012) Inactivation of ribosomal protein genes in *Bacillus subtilis* reveals importance of each ribosomal protein for cell proliferation and cell differentiation. J Bacteriol 194: 6282-6291.
51. Berkmen MB, Grossman AD (2007) Subcellular positioning of the origin region of the *Bacillus subtilis* chromosome is independent of sequences within *oriC*, the site of replication initiation, and the replication initiator DnaA. Mol Microbiol 63: 150-165.
52. Kadoya R, Hassan AK, Kasahara Y, Ogasawara N, Moriya S (2002) Two separate DNA sequences within *oriC* participate in accurate chromosome segregation in *Bacillus subtilis*. Mol Microbiol 45: 73-87.
53. Youngman PJ, Perkins JB, Losick R (1983) Genetic transposition and insertional mutagenesis in *Bacillus subtilis* with *Streptococcus faecalis* transposon Tn917. Proc Natl Acad Sci USA 80: 2305-2309.
54. Nanamiya H, Kasai K, Nozawa A, Yun CS, Narisawa T, et al. (2008) Identification and functional analysis of novel (p)ppGpp synthetase genes in *Bacillus subtilis*. Mol Microbiol 67: 291-304.
55. Mercier R, Kawai Y, Errington J (2013) Excess membrane synthesis drives a primitive mode of cell proliferation. Cell 152: 997-1007.
56. Wendrich TM, Marahiel MA (1997) Cloning and characterization of a *relA/spoT* homologue from *Bacillus subtilis*. Mol Microbiol 26: 65-79.
57. Cho E, Ogasawara N, Ishikawa S (2008) The functional analysis of YabA, which interacts with DnaA and regulates initiation of chromosome replication in *Bacillus subtils*. Genes Genetic Sys 83: 111-125.
58. Vagner V, Dervyn E, Ehrlich SD (1998) A vector for systematic gene inactivation in *Bacillus subtilis*. Microbiology (Reading, England) 144: 3097-3104.
59. Quisel JD, Burkholder WF, Grossman AD (2001) In vivo effects of sporulation kinases on mutant Spo0A proteins in *Bacillus subtilis*. J Bacteriol 183: 6573-6578.
60. Daniel RA, Harry EJ, Katis VL, Wake RG, Errington J (1998) Characterization of the essential cell division gene *ftsL(yIID)* of *Bacillus subtilis* and its role in the assembly of the division apparatus. Mol Microbiol 29: 593-604.
